# 5-Methyl etodesnitazene human metabolism: LC-ESI^±^-HRMS/MS analysis (mono- and di-protonation) of human hepatocyte incubations and positive biospecimens

**DOI:** 10.1007/s00216-026-06472-8

**Published:** 2026-04-28

**Authors:** Omayema Taoussi, Jeremy Carlier, Prince S. Gameli, Laura M. Huppertz, Giulia Bambagiotti, Francesco Tavoletta, Anastasio Tini, Diletta Berardinelli, Francesco P. Busardò, Volker Auwärter

**Affiliations:** 1 Department of Biomedical Sciences and Public Health, Marche Polytechnic University, Via Tronto, 10/a, 60126 Ancona, AN Italy; 2 Institute of Forensic Medicine, Forensic Toxicology, Medical Center, University of Freiburg, Faculty of Medicine, University of Freiburg, Freiburg, Germany; 3 Maria Cecilia Hospital, GVM Care & Research, Cotignola, Italy

**Keywords:** 5-Methyl etodesnitazene, Etomethazene, Nitazenes, Human metabolism, Di-protonation, Liquid chromatography-high-resolution mass spectrometry (LC-HRMS)

## Abstract

The rapid emergence of new synthetic opioids (NSOs), especially potent 2-benzylbenzimidazole derivatives known as nitazenes, poses challenges to public health and toxicologists. Among these substances, 5-methyl etodesnitazene has recently appeared on the illicit market. This compound was shown to be approximately twice as potent as fentanyl at the µ-opioid receptor, and overdose may lead to potentially fatal respiratory depression. Detecting the drug and its metabolites is therefore critical for clinical and forensic investigations. This study aimed to characterize the metabolic profile of 5-methyl etodesnitazene in humans. Metabolic transformations were investigated through incubations with ten-donor pooled human hepatocytes and analysis of blood and urine samples from a postmortem case positive for 5-methyl etodesnitazene. Samples were analyzed using liquid chromatography-electrospray ionization-high-resolution tandem mass spectrometry (LC-ESI-HRMS/MS) in positive- and negative-ionization modes, and software-aided data mining. In positive mode, 5-methyl etodesnitazene produced an intense signal in its di-protonated form, which generated informative spectra, facilitating structural elucidation. Biotransformations included *N*- and *O*-deethylation, consistent with structural analogs, as well as ω-carboxylation specific to the methylated benzimidazole core; phase II metabolites were not detected. We suggest *N*-deethyl-5-methyl etodesnitazene, *N*-deethyl-5-carboxy etodesnitazene, and 5-carboxy etodesnitazene in blood, and *N*-deethyl-5-carboxy-4′-hydroxy etodesnitazene, 5-carboxy-4′-hydroxy etodesnitazene, and *N*-deethyl-5-carboxy etodesnitazene in urine, as metabolite biomarkers of consumption in clinical and forensic settings. Overall, metabolite profiles were consistent across in vitro and in vivo data, with minor matrix-specific differences. Considering that nitazene metabolites are often active and their metabolism is susceptible to genetic polymorphism, further research on the pharmacokinetics and pharmacodynamics of 5-methyl etodesnitazene is warranted.

**Supplementary Information:**

The online version contains supplementary material available at 10.1007/s00216-026-06472-8.

## Introduction

The spread and abuse of new psychoactive substances (NPS) have been a challenge for health and law enforcement authorities for almost two decades as they try to curb their proliferation. Currently, more than 1200 NPS have been reported in over 120 countries worldwide between 2013 and 2024, according to the United Nations Office on Drug and Crime Early Warning System (UNODC, EWS) [1]. Of particular interest is the fast-paced evolution of the new synthetic opioid (NSO) agonists, which are NPS emulating the recreational effects of morphine and heroin, following the recent efforts to regulate fentanyl and its analogs in the United States and China [2–4]. Particularly, the re-surfacing of 2-benzylbenzimidazole opioids or “nitazenes”, molecules synthesized in the 1970 s and never marketed due to their high potency and limited therapeutic index, and the synthesis of other new analogs have largely contributed to the transformation of the NSO market since 2019 [5–11]. Nitazenes are commercialized as a cheap substitute for heroin or fentanyl and have been found in combination with benzodiazepines (“benzo-dope”) or sedatives (“tranq-dope”) to reduce manufacturing costs or enhance the effects of one another, causing debilitating health consequences and fatalities to naive users [12–14]. More recently it was found as a constituent of “Kush”, a Central African local drug typically consisting of cannabis extracts enriched with synthetic drugs and allegedly containing pulverized human remains, which has attracted national attention in Sierra Leone and other Western African countries [15].

**Fig. 1:**
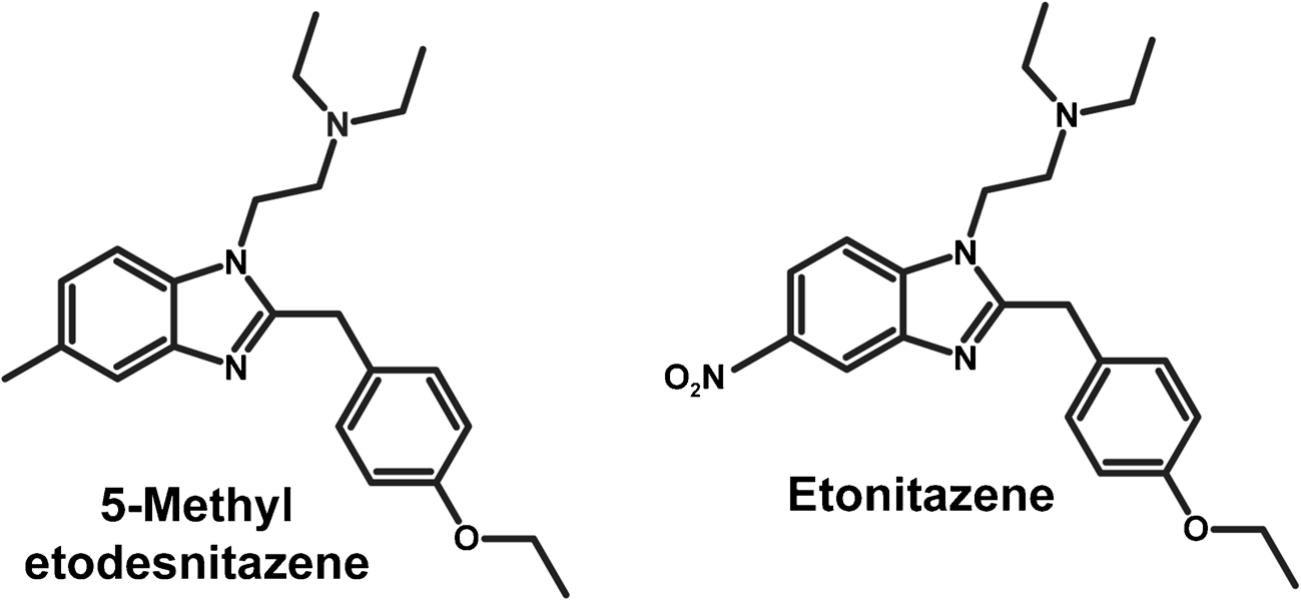
Chemical structures of 5-methyl etodesnitazene and etonitazene

5-Methyl etodesnitazene (2-[(4′-ethoxyphenyl)methyl]-*N*,*N*-diethyl-5-methyl-1*H*-benzimidazole-1-ethanamine) also known as etomethazene, is a 5-methyl analog of the highly potent etonitazene and recently emerged on the illicit drug market. The structures of 5-methyl etodesnitazene and etonitazene are shown in Fig. 1. 5-Methyl etodesnitazene was first reported in Latvia and by customs authorities in the United States in 2022, in Baltic and Scandinavian states in 2023, and in Germany and Poland in 2024 [16–20]. Two intoxication cases have been reported in the United States, but no data are available regarding the concentrations of the drug in biological matrices [21]. However, 5-methyl etodesnitazene was shown to be a full µ-opioid receptor agonist with high in vitro affinity (inhibition constant K_i_, 0.69 nmol/L) and potency (half-maximal effective concentration EC_50_, 10.5 nmol/L), values in a similar range to those of fentanyl (Ki = 9.78 nmol/L, EC_50_ = 17 nmol/L) [22], while showing limited effect at the κ- and δ-opioid receptors, suggesting that the drug may potentially induce strong analgesia and euphoria, but may also be responsible for potentially fatal respiratory depression and physical dependence [23]. Benzylbenzimidazole opioids such as metonitazene, which present an in vitro pharmacological profile similar to that of 5-methyl etodesnitazene [23], are typically detected at low concentrations in biological matrices due to their extensive hepatic metabolism and low active concentrations [12]. Characterizing 5-methyl etodesnitazene pharmacokinetics and identifying specific metabolite markers of consumption, which might be detected at higher concentrations and for extended time periods in biological matrices, is therefore relevant for proving exposure to provide suitable treatments, elucidating intoxications, and enhancing abstinence testing [5–7, 24–26]. However, 5-methyl etodesnitazene pharmacokinetics are currently unknown.

The present study aims to assess 5-methyl etodesnitazene human metabolism as a first step in the exploration of the drug’s pharmacokinetics and to identify specific consumption markers in order to enable detection of exposure in clinical and forensic casework. Human hepatocyte incubations and positive blood and urine samples from a forensic case were analyzed by liquid chromatography tandem high resolution mass spectrometry (LC-HRMS/MS) and software-assisted data mining as described below.

## Materials and methods

### Chemicals and reagents

LC-MS grade acetonitrile, water, methanol, and formic acid were obtained from Carlo Erba (Cornaredo, Italy). 5-Methyl etodesnitazene and diclofenac standards were purchased from Cayman Chemical (Ann Arbor, MI, USA) and Sigma Aldrich (Milan, Italy), respectively. Stock solutions of the standards were prepared to 1 mg/mL in methanol and stored at −20 °C prior to analysis.

Williams’ medium E, HEPES buffer (2-[4-(2-hydroxyethyl)−1-piperazinyl]ethanesulfonic acid), *l*-glutamine, ammonium acetate and β-glucuronidase were purchased from Sigma Aldrich. Supplemented Williams’ Medium E (SWM) was prepared by dissolving HEPES and L-glutamine at 2 and 20 mmol/L, respectively, in Williams’ medium E and the solution was stored at 4 °C until incubation. Thawing medium and ten-donor-pooled cryopreserved human hepatocytes were obtained from Lonza (Basel, Switzerland).

### In silico metabolites prediction

5-Methyl etodesnitazene human metabolites were predicted with the online GLORYx freeware (University of Hamburg, Germany) [27–30]. The metabolite list was generated using 5-methyl etodesnitazene SMILES (simplified molecular-input line-entry system) code generated through ChemSketch (Advanced Chemistry Development, Inc.; v. 2020.1.2) and selecting the “phase I and phase II metabolism” option. Metabolites with scores equal to or higher than 25% were selected and reprocessed to simulate a second metabolism step; the second-generation metabolite score was multiplied by the first-generation metabolite score. Metabolites with a prediction score equal to or higher than 25% were added to the LC-HRMS/MS inclusion list and the corresponding metabolic transformations were added to a list of predicted transformations for data mining.

### Hepatocyte incubation

Briefly, as previously detailed [29, 31, 32], hepatocytes were thawed in 50 mL thawing medium at 37 °C, then resuspended in SWM at 37 °C to reach a concentration of 2 × 10^6^ viable cells/mL. In sterile 24-well culture plates, 250 μL of hepatocyte suspension was gently mixed with 250 μL of 20 μmol/L 5-methyl etodesnitazene in SWM. The plate was incubated at 37 °C for 3 h, then reactions were quenched with 500 μL ice-cold acetonitrile followed by centrifugation at 15,000 × g for 10 min. The incubates were then stored at −80 °C until analysis. Negative controls included hepatocytes in SWM without the drug and the drug alone in SWM, both incubated for 0 and 3 h under identical conditions. Diclofenac was incubated under the same conditions to ensure metabolic activity of the cells.

### Positive real samples

Biological samples were obtained from a postmortem case. Samples resulted positive only for 5-methyl etodesnitazene, suggesting intoxication after drug ingestion. All samples were fully anonymized prior to analysis. No other substances of toxicological interest were detected.

### Sample preparation

#### Hepatocytes

The extraction of hepatocyte samples, as previously published [25, 29, 32], was as follows: after thawing at room temperature, incubates were centrifuged at 15,000 × g for 10 min. A 100 μL aliquot of the supernatant was mixed with 100 μL acetonitrile and centrifuged again under the same conditions. The liquid phase was evaporated to dryness under nitrogen at 37 °C and reconstituted in 100 μL water:acetonitrile 90:10 (v/v) containing 0.1% formic acid. After centrifugation under the same conditions, the supernatant was transferred into a vial with insert.

#### Urine

After thawing at room temperature, 100 µL urine was mixed with 200 µL of acetonitrile and centrifuged at 15,000 × g for 10 min [25, 26, 32]. The supernatant was evaporated to dryness under nitrogen at 37 °C and reconstituted in 100 µL water:acetonitrile 90:10 (v/v) containing 0.1% formic acid. After centrifugation under the same conditions, the supernatant was transferred into a vial with insert.

Additionally, 100 µL urine was mixed with 10 µL of 10 mol/L ammonium acetate (pH 5.0) and 100 µL of β-glucuronidase (5000 units) in conical glass tubes. After 90 min incubation at 37 °C, the sample was mixed with 400 µL ice-cold acetonitrile and centrifuged at 15,000 × g for 10 min. The supernatant was evaporated to dryness under nitrogen at 37 °C and reconstituted in 100 µL water:acetonitrile 90:10 (v/v) containing 0.1% formic acid. After centrifugation under the same conditions, the supernatant was transferred into a vial with insert. A control was prepared simultaneously under the same conditions using 100 µL of water instead of β-glucuronidase.

### Instrumental conditions

LC-HRMS/MS analysis was carried out with a DIONEX UltiMate 3000 liquid chromatograph coupled with a Q-Exactive quadrupole-Orbitrap hybrid high-resolution mass spectrometer with a heated-electrospray-ionization source (Thermo Scientific; Waltham, MA, USA).

#### Liquid chromatography conditions

As previously described for similar method [25, 33], a volume of 10 μL sample was injected into the LC-HRMS/MS system. A Kinetex® Biphenyl column (150 × 2.1 mm, 2.6 μm) from Phenomenex (Torrance, CA, USA) was used and maintained at 37 °C throughout the separation. The mobile phases consisted of 0.1% formic acid in water as mobile phase A (MPA) and 0.1% formic acid in acetonitrile as mobile phase B (MPB). The gradient elution started with 5% MPB held for 2 min, increased to 25% MPB over 14 min, further increased to 50% MPB in 5 min, then ramped up to 95% MPB within 1 min and maintained for 5 min. The initial conditions (5% MPB) were restored within 0.1 min and maintained until the end of the 30 min run. The flow rate was 0.4 mL/min.

#### Mass spectrometry conditions

Before the analysis, ionization source settings were optimized after injecting 5-methyl etodesnitazene standard at 1 μg/mL in MPA:MPB 90:10 (v/v). Investigations were performed in positive- (two injections with two different inclusion lists) and negative-ionization modes (one injection), and HESI parameters were the same for both modes: 50 a.u. for sheath-gas flow rate, 10 a.u. for auxiliary-gas flow rate, ± 3,5 kV for spray voltage, 300 °C for capillary- and auxiliary-gas heater temperature, and 50 a.u. for S-lens radio frequency; sweep gas was not applied. The orbitrap was calibrated prior to analysis, and the method used a lock mass list for internal calibration during each injection.

Data was acquired from 1 to 25 min of the chromatographic run in full-scan HRMS (FullMS) and data-dependent MS/MS (ddMS^2^) mode. The FullMS acquisition range was *m/z* 100–700 with a resolution of 70,000 at full width at half maximum at *m/z* 200; automatic gain control (AGC) target was 3 × 10^6^ and maximum injection time (IT) 200 ms. A maximum number of five ddMS^2^ scans were triggered at each cycle with a dynamic exclusion of 2.0 s and an intensity threshold of 10^4^ for each FullMS scan depending on an inclusion based on the in silico predictions and postulation [25] (Supplementary Table S1). Due to 5-methyl etodesnitazene susceptibility to protonation in positive-ionization mode, the 3 h incubate with hepatocytes was reinjected with two different inclusion lists using the theoretical *m/z* of single- or double-protonated putative metabolites to increase the chances to obtain non-interfered HRMS/MS spectra. Ions that were not included in the inclusion list also triggered ddMS^2^ scans, although they were not priority (“pick others if idle” option). Additionally, background ions were compiled in an exclusion list to avoid unnecessary fragmentation. The ddMS^2^ isolation window was *m/z* 1.2 with a resolution of 17,500, and the normalized collision energy (NCE) was 40, 55, and 80 a.u; AGC target was 2 × 10^5^ and maximum IT was 64 ms.

### Metabolites identification in hepatocyte incubations and urine samples

LC-HRMS data were processed using Compound Discoverer (v. 3.1.1.12) from Thermo Scientific, employing a partially automated method [26, 31] with minor modifications. Briefly, ions detected in HRMS were compared to a theoretical metabolite list derived from phase I and phase II transformations, based on in silico predictions; the list of transformations is detailed in Supplementary Table S2 (intensity threshold, 5 × 10^3^; mass tolerance, 5 ppm). The HRMS/MS spectra and theoretical elemental compositions of the ions were matched against mzCloud (Counterfeit Drug (Therapeutic), Drugs of Abuse/Illegal Drugs, and Therapeutics/Prescription Drugs libraries) and ChemSpider (Cayman Chemical and DrugBank libraries) online databases (intensity threshold, 10^5^; HRMS mass tolerance, 5 ppm; HRMS/MS mass tolerance, 10 ppm). Chromatographic peaks detected in control samples with similar or higher intensity than those in the experimental samples were flagged for manual inspection.

## Results

### In silico prediction of 5-methyl etodesnitazene metabolites

Nine first-generation metabolites (pM_1_–pM_9_, ranked by descending score) and 45 s-generation metabolites (pM_X-1_-pM_X-11_, where pM_X_ is the corresponding first-generation metabolite) were predicted; 19 s-generation metabolites were duplicates (Supplementary Table S3). Phase I reactions such as hydroxylation/oxidation, 5-methyl carboxylation, *N*-deethylation, *O*-deethylation, and oxidative deamination were predicted. Phase II *O*-glucuronidation and *O*-sulfation were predicted with a high score when following hydroxylation, carboxylation, *O*-dealkylation, and oxidative deamination. Distinct predicted metabolites, representing unique molecular entities generated from different transformation pathways, were incorporated into a ddMS^2^ inclusion list for LC-HRMS/MS analysis (“Mass spectrometry conditions”) and a list of potential reactions for automated data mining (“Metabolites identification in hepatocyte incubations and urine samples”).

### 5-Methyl etodesnitazene fragmentation pattern

**Fig. 2:**
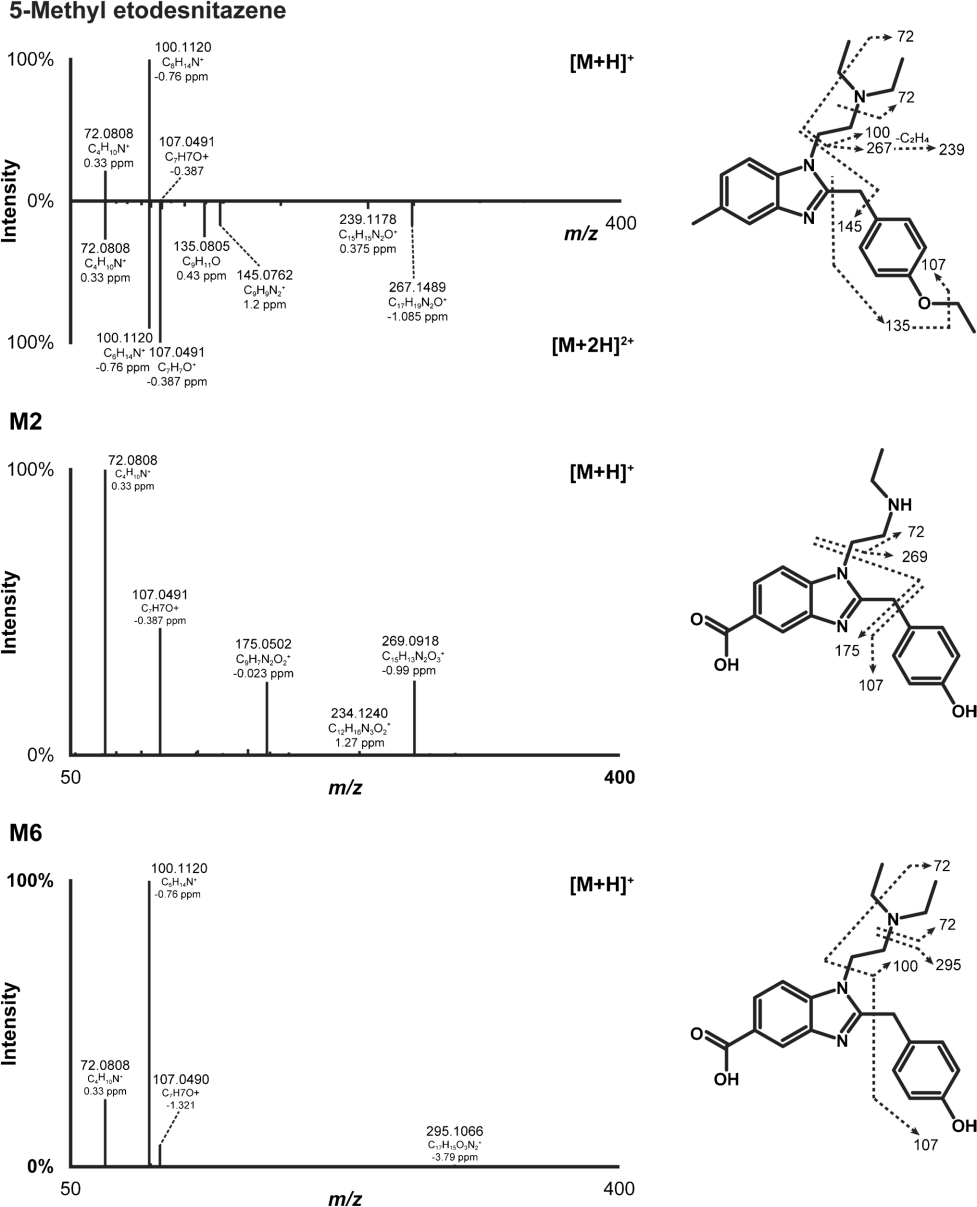
High-resolution tandem mass spectrometry spectra after positive electrospray ionization and suggested fragmentation of 5-methyl etodesnitazene and major metabolites M2 and M6; [M+H]^+^, single protonation; [M+2H]^2+^, double protonation

5-Methyl etodesnitazene was only detected in positive-ionization mode under the present analytical conditions. In this article, LC-HRMS intensities and fragmentation patterns are described following positive electrospray ionization, unless specified otherwise. 5-Methyl etodesnitazene eluted at 10.42 min of the chromatographic run with a signal at *m/z* 366.2534 (monoprotonated molecule, [M+H]^+^) and 183.6305 (diprotonated molecule, [M+2H]^2+^), as shown in Fig. 2. The monoprotonated molecule of 5-Methyl etodesnitazene was fragmented following a similar pattern to that of etodesnitazene under the same analytical conditions [25] (Fig. 2). The most intense fragments correspond to the *N*,*N*-diethylethanamine (*m/z* 100.1120, C_6_H_14_N^+^) and diethylamine (*m/z* 72.0808, C_4_H_10_N^+^) side chains, and the fragment at *m/z* 107.0491 corresponds to the 1′-methyl-4′-hydroxybenzyl group (C_7_H_7_O^+^). The fragmentation spectrum generated from the diprotonated ion displayed additional major fragments at *m/z* 267.1489 produced by *N*,*N*-diethylethanamine loss (C_17_H_19_N_2_O^+^), which was further fragmented to *m/z* 239.1174 after ethyl loss (C_15_H_15_N_2_O^+^); the fragments at *m/z* 145.0760 and 135.0802 correspond to the 2,5-dimethyl-benzimidazole (C_9_H_9_N_2_^+^) and the 1′-methyl-4′-ethoxybenzene group (C_9_H_11_O^+^), respectively.

### Metabolite identification in human hepatocyte incubations

**Fig. 3:**
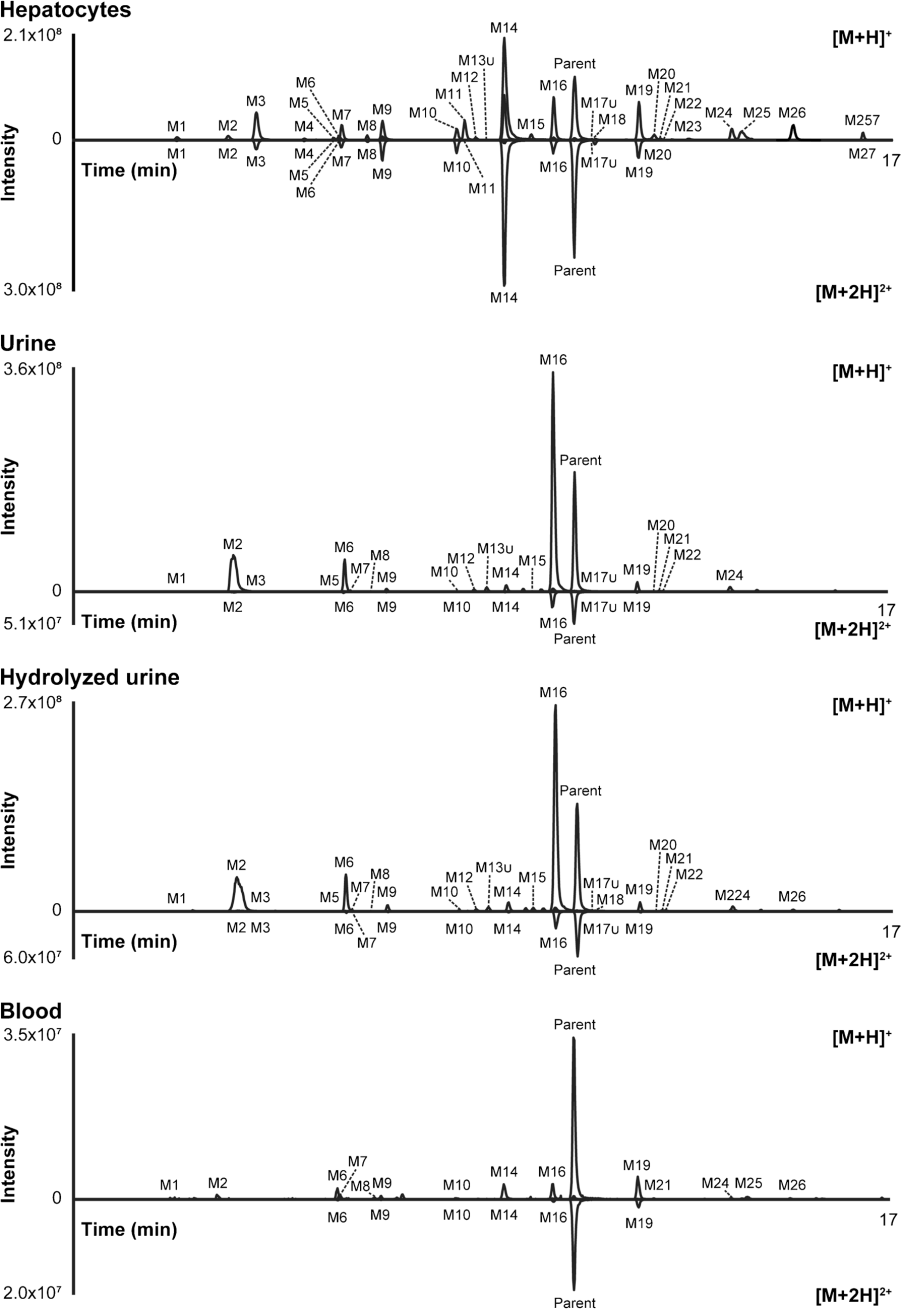
Extracted-ion chromatograms in positive-ionization mode of 5-methyl etodesnitazene and metabolites after incubation of the substance with 10-donor-pooled human hepatocytes and in an authentic 5-methyl etodesnitazene-positive case (urine, urine with glucuronide hydrolysis, and blood). Mass tolerance, 5 ppm; [M+H]^+^, single protonation; [M+2H]^2+^, double protonation

Twenty-five 5-methyl etodesnitazene metabolites were identified in hepatocyte incubates; they consistently produced higher signal intensity in positive-ionization mode. Analysis in negative ionization mode did not detect any metabolites. The metabolites were named M1 to M27 by ascending retention time, with two metabolites (M13u and M17u) identified retrospectively rather than being directly detected. 5-Methyl etodesnitazene mono- and di-protonated molecules produced an LC-HRMS peak areas of 4.1 × 10^9^ and 1.2 × 10^10^ a.u., respectively, in hepatocyte incubates at T_0h_. The extracted-ion chromatogram of 5-methyl etodesnitazene and its metabolites identified after 3 h of incubation is displayed in Fig. 3.

**Fig. 4:**
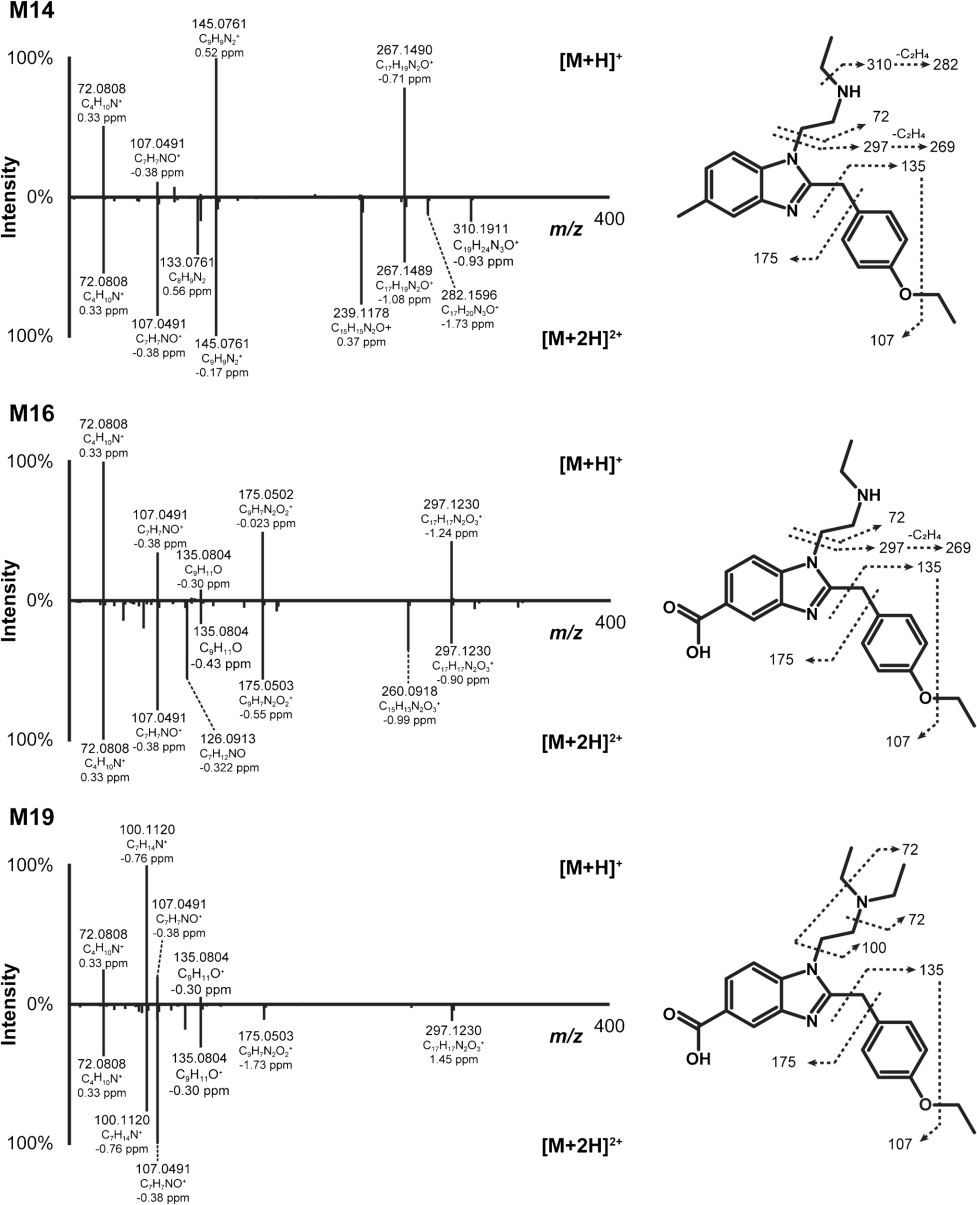
High-resolution tandem mass spectrometry spectra after positive electrospray ionization and suggested fragmentation of 5-methyl etodesnitazene major metabolites M14, M16, and M19; [M+H]^+^, single protonation; [M+2H]^2+^, double protonation

The most intense metabolites were formed via *N*-deethylation, *O*-deethylation, and/or 5-methyl carboxylation. Additional reactions included hydroxylation, oxidation to carbonyl group, oxidative deamination, and combinations of these. Table 1 presents the elemental composition, retention time, accurate mass of mono- and di-protonated molecules, and LC-HRMS peak areas of 5-methyl etodesnitazene and its metabolites in positive-ionization mode after 3 h of incubation with hepatocytes. The fragmentation patterns of the major 5-methyl etodesnitazene metabolites in positive-ionization mode are shown in Figs. 2 and 4, while those of the remaining metabolites are presented in Supplementary Materials.

**Table 1:** Metabolic transformation, elemental composition, retention time (RT), accurate mass of molecular ion, deviation from theoretical accurate mass, and liquid chromatography-high-resolution mass spectrometry peak area of 5-methyl etodesnitazene and metabolites in positive ionization mode after 3 h Incubation with human hepatocytes and in postmortem samples. Mass tolerance, 5 ppm. *hydro*, β-glucuronide hydrolysis; *ND*, not detected

ID	Biotransformation	Elemental composition	RT (min)	*m/z* (Δppm): [M+H]^+^ [M+2H]^2+^	Peak area in incubates: [M+H]^+^ [M+2H]^2+^	Peak area in real case: [M+H]^+^ [M+2H]^2+^		
						**Urine** **no hydro**	**Urine** **hydro**	**Blood**
M1	*O*-Deethylation + Hydroxylation	C_21_H_27_N_3_O_2_	2.15	354.2172 (−1.1) 177.6123 (−0.8)	3.0 × 10^7^ 8.0 × 10^6^	1.8 × 10^6^ ND	4.9 × 10^6^ ND	4.7 × 10^5^ ND
M2	*N*-Deethylation + *O*-Deethylation + ω-Carboxylation	C_19_H_21_N_3_O_3_	3.22	340.1652 (−1.1) 170.5864 (−0.1)	4.8 × 10^7^ 2.9 × 10^6^	5.7 × 10^8^ 9.1 × 10^6^	5.0 × 10^8^ 9.9 × 10^6^	3.8 × 10^6^ ND
M3	*N*-Deethylation + *O*-Deethylation	C_19_H_23_N_3_O	3.80	310.1909 (−1.6) 155.5992 (−0.8)	3.4 × 10^8^ 1.0 × 10^8^	7.6 × 10^5^ ND	2.5 × 10^6^ 3.6 × 10^5^	ND ND
M4	*N*-Deethylation + Hydroxylation (ethoxyphenyl)	C_21_H_27_N_3_O_2_	4.80	354.2172 (−1.1) 177.6122 (−1.3)	1.3 × 10^7^ 8.9 × 10^6^	ND ND	ND ND	ND ND
M5	*N*-Deethylation + *N*-Deethylation + Hydroxylation (methylbenzimidazole)	C_19_H_23_N_3_O_2_	5.41	326.1859 (−1.2) 163.5968 (+ 0.1)	1.8 × 10^7^ 1.0 × 10^5^	2.5 × 10^6^ ND	2.4 × 10^6^ ND	ND ND
M6	ω-Carboxylation + *O*-Deethylation	C_21_H_25_N_3_O_3_	5.51	368.1964 (−1.3) 184.6018 (−1.5)	3.5 × 10^7^ 6.3 × 10^6^	1.9 × 10^8^ 6.4 × 10^6^	1.8 × 10^8^ 3.7 × 10^6^	8.6 × 10^6^ 7.9 × 10^5^
M7	*O*-Deethylation	C_21_H_27_N_3_O	5.56	338.2221 (−1.7) 169.6150 (+ 0.1)	1.3 × 10^8^ 6.3 × 10^7^	1.7 × 10^6^ ND	7.9 × 10^6^ 1.9 × 10^6^	4.1 × 10^6^ ND
M8	Hydroxylation (ethoxyphenyl) + *O*-Demethylation	C_22_H_29_N_3_O_2_	6.11	368.2328 (−1.2) 184.6201 (−0.9)	2.4 × 10^7^ 1.4 × 10^7^	1.4 × 10^5^ ND	2.0 × 10^6^ ND	2.3 × 10^5^ ND
M9	*N*-Deethylation + Hydroxylation (methylbenzimidazole)	C_21_H_27_N_3_O_2_	6.43	354.2171 (−1.4) 177.6123 (−0.8)	1.8 × 10^8^ 1.6 × 10^8^	1.6 × 10^7^ 2.4 × 10^6^	3.0 × 10^7^ 3.4 × 10^6^	2.2 × 10^6^ 4.0 × 10^5^
M10	Hydroxylation (methylbenzimidazole)	C_23_H_31_N_3_O_2_	7.97	382.2485 (−1.1) 191.6280 (−0.5)	1.0 × 10^8^ 1.1 × 10^8^	6.1 × 10^6^ 8.9 × 10^5^	5.8 × 10^6^ 1.0 × 10^6^	8.7 × 10^5^ 3.4 × 10^5^
M11	*N*-Deethylation + *N*-Deethylation	C_19_H_23_N_3_O	8.13	310.1909 (−1.6) 155.5993 (−0.2)	1.7 × 10^8^ 1.0 × 10^6^	ND ND	ND ND	ND ND
M12	Oxidative deamination + *O*-Deethylation + Oxidation to ketone group (methyl linker)	C_17_H_16_N_2_O_3_	8.36	297.1229 (−1.6) ND	2.1 × 10^7^ ND	1.5 × 10^7^ ND	1.2 × 10^7^ ND	ND ND
M13_U_	*N*-Deethylation + *N*-Deethylation + ω-Carboxylation + O-Demethylation	C_19_H_21_N_3_O_3_	8.59	340.1652 (−1.1) ND	4.6 × 10^6^ ND	2.8 × 10^7^ ND	2.5 × 10^7^ ND	ND ND
M14	*N*-Deethylation	C_21_H_27_N_3_O	8.96	338.2223 (−1.1) 169.6150 (+ 0.1)	1.3 × 10^9^ 1.4 × 10^9^	4.4 × 10^7^ 3.4 × 10^6^	5.2 × 10^7^ 5.1 × 10^6^	1.5 × 10^7^ 4.2 × 10^6^
M15	Oxidative deamination + *O*-Deethylation	C_17_H_18_N_2_O_2_	9.52	283.1439 (−0.7) ND	5.0 × 10^7^ ND	2.9 × 10^6^ ND	1.8 × 10^7^ ND	ND ND
M16	*N*-Deethylation + ω-Carboxylation	C_21_H_25_N_3_O_3_	9.98	368.1963 (1.5) 184.6018 (−1.5)	3.8 × 10^8^ 1.1 × 10^8^	1.6 × 10^9^ 1.0 × 10^8^	1.3 × 10^9^ 1.0 × 10^8^	1.3 × 10^7^ 2.1 × 10^6^
**5-Methyl etodesnitazene**		**C**_**23**_**H**_**31**_**N**_**3**_**O**	**10.42**	**366.2534 (−1.6)** **183.6305 (−0.7)**	**7.0 × 10**^**8**^ **9.7 × 10**^**8**^	**8.1 × 10**^**8**^ **2.2 × 10**^**8**^	**6.7 × 10**^**8**^ **2.6 × 10**^**8**^	**1.5 × 10**^**8**^ **8.4 × 10**^**7**^
M17_U_	*O-*Deethylation + ω-Carboxylation + Desaturation	C_21_H_23_N_3_O_3_	10.84	366.1808 (−1.1) 183.5941 (−0.8)	6.8 × 10^6^ 8.1 × 10^5^	1.0 × 10^8^ 2.8 × 10^6^	8.8 × 10^7^ 3.1 × 10^6^	ND ND
M18	Oxidative deamination + Hydroxylation (methyl linker)	C_19_H_22_N_2_O_3_	10.86	327.1699 (−1.3) ND	1.0 × 10^7^ ND	ND ND	1.9 × 10^6^ ND	ND ND
M19	ω-Carboxylation	C_23_H_29_N_3_O_3_	11.76	396.2277 (−1.2) 198.6176 (−0.6)	3.5 × 10^8^ 1.7 × 10^8^	5.2 × 10^7^ 4.4 × 10^6^	4.0 × 10^7^ 4.7 × 10^6^	2.0 × 10^7^ 7.5 × 10^6^
M20	*N*-Deethylation + Oxidation to ketone group (methyl linker)	C_21_H_25_N_3_O_2_	12.07	352.2018 (−0.4) 176.6046 (−0.1)	7.1 × 10^8^ 2.4 × 10^6^	5.3 × 10^5^ ND	6.6 × 10^6^ ND	ND ND
M21	*N*-Deethylation + ω-Carboxylation + Oxidation to ketone group (*N*-ethyl)	C_21_H_23_N_3_O_4_	12.12	382.1757 (−1.1) ND	2.1 × 10^7^ ND	2.6 × 10^7^ ND	2.2 × 10^7^ ND	3.7 × 10^5^ ND
M22	Oxidative deamination + Hydroxylation + Oxidation to ketone group (methyl linker)	C_19_H_20_N_2_O_4_	12.20	341.1496 (+ 0.0) ND	1.4 × 10^7^ ND	1.3 × 10^7^ ND	1.2 × 10^7^ ND	ND ND
M23	*N*-Deethylation + *N*-Deethylation + Hydroxylation *(N*-ethylamine)	C_19_H_23_N_3_O_2_	12.79	326.1860 (−0.9) ND	2.3 × 10^7^ ND	ND ND	ND ND	ND ND
M24	Oxidative deamination + Oxidation to ketone group	C_19_H_20_N_2_O_3_	13.69	325.1543 (−1.1) ND	1.2 × 10^8^ ND	3.9 × 10^7^ ND	3.3 × 10^7^ ND	1.3 × 10^6^ ND
M25	Oxidative deamination + Hydroxylation	C_19_H_22_N_2_O_3_	13.90	327.1700 (−1.0) ND	1.5 × 10^8^ ND	ND ND	ND ND	1.2 × 10^6^ ND
M26	Oxidative deamination	C_19_H_22_N_2_O_2_	14.92	311.1750 (−1.3) ND	1.3 × 10^8^ ND	ND ND	1.0 × 10^7^ ND	9.8 × 10^5^ ND
M27	*N*-Deethylation + Hydroxylation (*N*-ethyl)	C_21_H_27_N_3_O_2_	16.42	354.2172 (−1.1) 177.6123 (−0.8)	5.5 × 10^7^ 3.2 × 10^5^	ND ND	ND ND	ND ND

### Metabolite identification in urine and blood samples

Twenty-one out of 25 metabolites identified in vitro were also detected in human samples, and 2 additional minor phase I metabolites, M13_U_ and M17_U_, were detected in hydrolyzed and non-hydrolyzed urine. All metabolites identified in hepatocyte incubates beside M4, M11, M23, M25, and M27 were detected in urine with or without glucuronide hydrolysis; 5-methyl etodesnitazene signal intensity was only second to M16, with or without hydrolysis. M1, M2, M6–10, M14, M16, M18, M21, and M24–26 were detected in blood; the parent signal was largely predominant.

Consistent with in vitro results, the main metabolic transformations were *N*-deethylation, *O*-deethylation, and/or 5-methyl carboxylation in urine and *N*-deethylation and/or 5-methyl carboxylation in blood. Table 1 provides detailed information on the elemental composition, retention time, accurate molecular ion mass, and LC-HRMS peak area for 5-methyl etodesnitazene and its metabolites, analyzed in positive-ionization mode from real samples. Peak areas reported in Table 1 only reflect relative signal abundance within the same matrix and analytical run. The chromatograms of the extracted ions for 5-methyl etodesnitazene and metabolites in urine (with and without hydrolysis) and in blood are presented in Fig. 3. The structure elucidation process of 5-methyl etodesnitazene main metabolites is described below.

#### ω-Carboxylation (M19 structure elucidation)

M19 was the most intense metabolite in the blood sample and was also detected in urine, albeit at a lower level. The metabolite eluted at 11.76 min, i.e., after 5-methyl etodesnitazene and was formed by carboxylation, as suggested by the + 29.9743 Da (−2H+2O) shift from the parent compound (mono-protonated forms). M19 yielded the fragments at *m/z* 72.0808, 100.1120, 107.0491, and 135.0804, which were also observed for the parent, suggesting that the side chains remained unchanged. The fragmentation of the di-protonated ion produced an additional ion at *m/z* 175.0498, corresponding to the 2,5-dimethyl-benzimidazole core with the removal of 2H and the addition of 2O (C_9_H_7_N_2_O_2_^+^), confirming that the transformation occurred at this moiety and implying carboxylation of the methyl group.

#### N-Deethylation (M14 structure elucidation)

M14 was predominant in vitro and was the second most intense metabolite in the 5-methyl etodesnitazene-positive blood. Deethylation (–C_2_H_4_) was suggested by a −28.0311 Da shift from the parent compound and a retention time of 8.96 min. M14 fragmentation pattern included ions at *m/z* 145.0761 and 267.1490, which are also observed for the parent compound, indicating that the transformation occurred at the *N,N*-diethylethanamine side chain. The presence of an intense fragment at *m/z* 72.0808 (diethylamine, C_4_H_10_N^+^) and the absence of any ion at the theoretical accurate mass of the *N,N*-diethylethanamine (*m/z* 100.1121 ± 5 ppm, C_6_H_14_N^+^) further supported an *N*-deethylation. Additional fragments were found upon fragmentation of the di-protonated M14 ion at *m/z* 310.1911 and 282.1596, corresponding to the loss of an ethyl (C_19_H_24_N_3_O^+^) and a di-ethyl group (C_17_H_20_N_3_O^+^), respectively. A fragment at *m/z* 239.1178 (C_15_H_15_N_2_O^+^) was also detected, corresponding to a secondary ethyl loss from the ion at *m/z* 267.1490.

#### ω-Carboxylation and N-deethylation (M16 structure elucidation)

A combination of carboxylation (−2H+2O) and deethylation (–C_2_H_4_) was identified in M16, as indicated by a + 1.9429 Da shift relative to the parent compound. Similar to M14, *N*-deethylation was supported by the presence of an intense fragment at *m/z* 72.0808 and the absence of a fragment with *m/z* 100.1121 (“N-Deethylation (M14 structure elucidation)”). Similar to M19, ω-carboxylation was evidenced by a delayed retention time compared to M14 (*N*-deethyl-5-methyl etodesnitazene) and the fragments at *m/z* 175.0502 and 297.1230 (“ω-Carboxylation (M19 structure elucidation)”). M16 was the metabolite with the most intense signal in urine.

#### ω-Carboxylation and O-deethylation (M6 structure elucidation)

M6 had the same elemental composition as M16 and was also produced by carboxylation (−2H+2O) and deethylation (–C_2_H_4_), as suggested by a + 1.9430 Da shift from the parent compound. The mono-protonated ion yielded only three fragments at *m/z* 72.0808, 100.1120, and 107.0491, which were also observed in 5-methyl etodesnitazene. Additionally, fragment m/z 295.1066, produced after the loss of the *N*,*N*-diethylamine group was detected with a very low signal.

The signal of the di-protonated ion was low and therefore did not produce additional relevant fragments for structure elucidation. Ions at *m/z* 72.0808 and 100.1120 indicated that the *N,N*-diethylethanamine side chain remained unchanged. The presence of a fragment at *m/z* 107.0490 and the absence of any ion at the theoretical accurate mass of the 1′-methyl-4′-ethoxybenzyl group (*m/z* 135.0804 ± 5 ppm, C_9_H_11_O^+^) further indicated that dealkylation occurred at the *O*-ethyl group, while carboxylation occurred at the methyl group of the 5-methyl-benzimidazole core. Notably, *O*-dealkylation substantially reduced M6 elution time, as previously shown during the LC-HRMS/MS analysis of structural analogs under the same conditions [25].

#### ω-Carboxylation, N-deethylation, and O-deethylation (M2 structure elucidation)

M2 resulted from a combination of carboxylation (−2H+2O) and di-deethylation [–(C_2_H_4_)_2_], as suggested by a −26.0882 Da shift relative to the parent compound. As for M14, *N*-deethylation was supported by the presence of an intense fragment at *m/z* 72.0808 and the absence of a fragment at *m/z* 100.1121 (“N-Deethylation (M14 structure elucidation)”). Similar to M19, ω-carboxylation was suggested by the fragment at *m/z* 175.0502 (“ω-Carboxylation (M19 structure elucidation)”). Like M6, *O*-deethylation was demonstrated by a short elution time, the presence of a fragment at *m/z* 107.0490, and the absence of a fragment with *m/z* 135.0804 (“ω-Carboxylation and O-deethylation (M6 structure elucidation)”).

## Discussion

### Mono- and di-protonation

Nitazenes carrying an *N*,*N*-diethylamine group typically yield few fragments at low *m/z* values. This is due to the presence of the tertiary aliphatic amine, which is the most basic site in the molecule and is readily protonated, producing predominantly diethylamine (*m/z* 72.0808, C_4_H_10_N^+^) and *N*,*N*-diethylethanamine (*m/z* 100.1121, C_6_H_14_N^+^) fragments [25, 34–37]. These few fragments are less specific than larger ones and therefore more prone to interferences; they also provide only limited information for the structural elucidation of metabolites, making their identification potentially challenging. Di-protonation provides better structural insight, as charge retention after amine cleavage allows the remaining molecular scaffold to remain detectable and generate richer fragmentation. Optimizing ESI settings and mobile phase conditions can favor di-protonation to increase fragment production or avoid potential interference at the mono-protonated *m/z*, although di-protonation also largely depends on the compound structure (e.g., size, number of protonation sites). 5-methyl etodesnitazene produces an intense signal for its di-protonated form. Compared to etodesnitazene, the methyl group at the 5-position of the benzimidazole core in 5-methyl etodesnitazene is an electron donor and might increase the electron density in the benzimidazole ring (inductive effect), thereby increasing the basicity and potential protonation of the nitrogen atoms of the imidazole ring. The di-protonated form of most metabolites also is intense, but seems substantially reduced by *N*,*N*-dideethylation and oxidative deamination at the aliphatic tertiary amine, as well as *O*-deethylation. In the present study, the samples were injected twice in positive-ionization mode with two different inclusion lists using the theoretical *m/z* of mono- or di-protonated putative metabolites to ensure the fragmentation of the two protonated forms. Source settings and LC conditions were not optimized to facilitate comparison with previous studies on the identification of metabolites of structural analogs [25, 26].

### 5-Methyl etodesnitazene metabolites

**Fig. 5:**
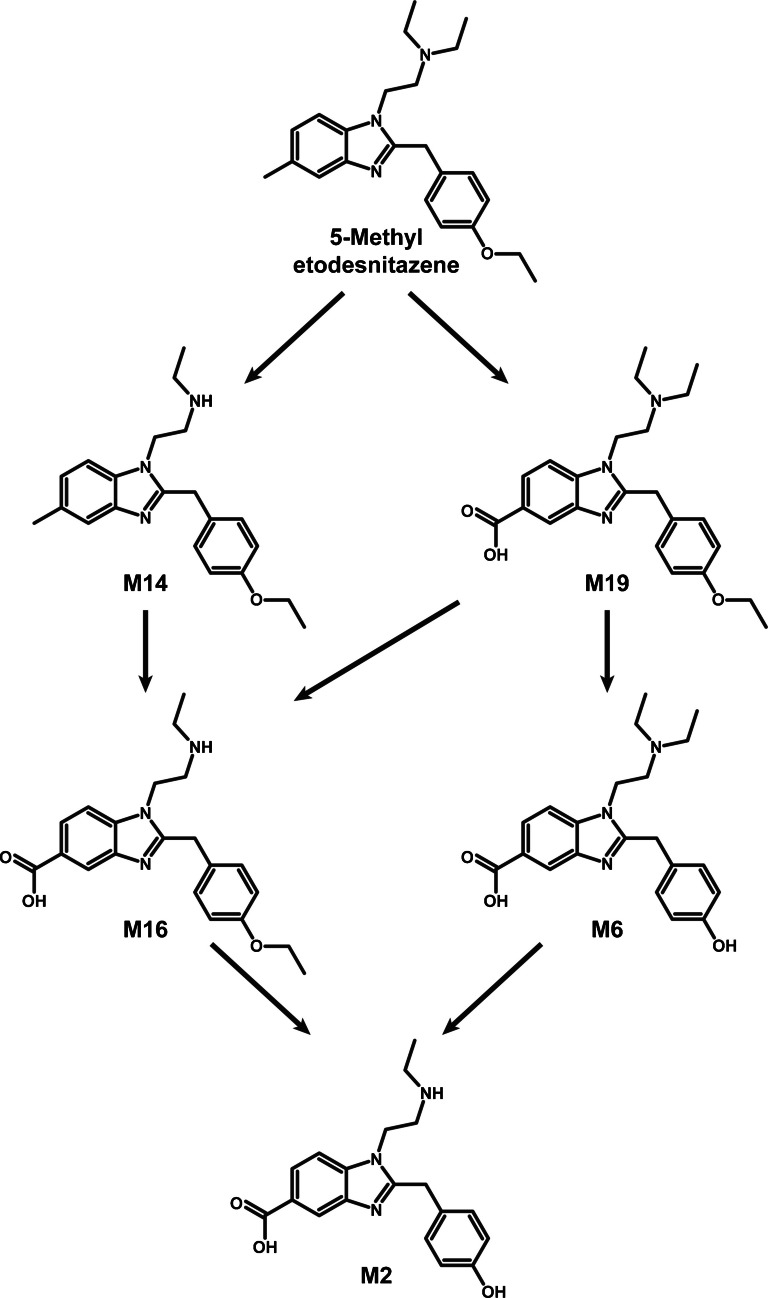
5-methyl etodesnitazene suggested metabolic fate in humans (only main metabolites)

The suggested metabolic fate of 5-methyl etodesnitazene in humans is illustrated in Fig. 5.

In the blood sample, the parent compound had a more intense signal compared to its metabolites, possibly indicating recent use. Nitazenes are also known to be metabolized by highly polymorphic enzymes, which may impact their metabolism across individuals [38]. Additional samples from other positive cases varying in dose and time of collection post-consumption should be analyzed to refine the present findings.

The transformations were the same as observed with etodesnitazene, with *N*- and *O*-deethylation being major reactions [25], but the presence of the 5-methyl group also induced the formation of ω-carboxy derivatives. Phase II metabolites were not detected, consistent with the in vivo metabolism of etodesnitazene, in which glucuronides were marginal [25]. Generally good congruence was found between human hepatocyte incubations and positive biological samples, with only two additional minor metabolites identified in urine, although M2 and M6 seem to be underrepresented in the in vitro experiment. Similar results were also found in urine and in blood. Interestingly, and in line with the metabolism of structural analogs, *O*-deethyl derivatives were predominant in urine, while *N*-deethyl metabolites were more prevalent in blood [25, 39, 40], which is likely due to a faster urinary elimination of *O*-dealkyl nitazenes. The metabolism of 5-methyl nitazenes has yet to be reported, and methyl carboxylation, which is particularly prevalent in blood, currently lacks a point of comparison.

5-Methyl etodesnitazene metabolites might participate to and prolongate the overall effects and toxicity of the drug, particularly the predominant metabolites identified in blood such as M16 (combination of *N*-deethylation and ω-carboxylation). *N*-Deethyl nitazene metabolites are typically active, and sometimes even more potent than the parent drug, as demonstrated in vitro µ-opioid receptor activation assays [5, 7, 34, 41]. While no data are currently available on the activity of metabolites carboxylated at the 5-methyl group, substitution at position 5 of the benzimidazole core is known to significantly influence activity, with the following order of potency: etonitazene (nitro) > 5-methyl etodesnitazene (methyl) > etodesnitazene (hydrogen) > 5-amino etodesnitazene (amine) [23, 42]. Further research is needed on the activity of 5-methyl etodesnitazene metabolites and the structure-activity relationships of nitazene derivatives.

Interestingly, some metabolites (M19–M25) showed higher retention times than the parent drug. While counterintuitive for traditional C18 phases, this is explained by the unique selectivity of the biphenylsiloxane-bonded stationary phase used. As described by Atapattu et al*. *[43], biphenyl phases exhibit high polarizability (s) and π-π interaction (e) constants. Biotransformations, such as ω-carboxylation, likely increase the dipole-dipole or π-π interactions with the phase. In these cases, such electronic interactions outweigh the increase in hydrophilicity, leading to greater retention compared to the parent 5-methyl etodesnitazene.

### Biomarkers of consumption

Major metabolites identified in blood and urine samples from 5-methyl etodesnitazene-positive individuals include *N*-deethyl-5-methyl etodesnitazene (M14), *N*-deethyl-5-carboxy etodesnitazene (M16), and 5-carboxy etodesnitazene (M19) in blood, and *N*-deethyl-5-carboxy-4′-hydroxy etodesnitazene (M2), 5-carboxy-4′-hydroxy etodesnitazene (M6), and *N*-deethyl-5-carboxy etodesnitazene (M16) in urine. These metabolites may serve as reliable biomarkers for 5-methyl etodesnitazene consumption. Considering the absence of Phase II metabolites, urine hydrolysis does not seem necessary to increase the signal of non-conjugated metabolites. To the best of the authors’ knowledge, these metabolites were not identified for other structural analogs, as all the metabolites identified in the present study contained the 5-methylbenzimidazole core of the molecule. The parent compound was also intense in both matrices and should also be targeted when attempting to prove consumption.

## Conclusions

The present study provides a detailed investigation of the metabolic fate of 5-methyl etodesnitazene using human hepatocyte incubations and 5-methyl etodesnitazene-positive blood and urine from a postmortem case. The compound underwent similar biotransformations to its analogs, including *N*- and *O*-deethylation, but also exhibits specific pathways such as ω-carboxylation at the methylated benzimidazole core. These reactions result in a distinctive metabolite profile, with *N*-deethyl and carboxylated derivatives emerging as major metabolites. We suggest *N*-deethyl-5-methyl etodesnitazene (M14), *N*-deethyl-5-carboxy etodesnitazene (M16), and 5-carboxy etodesnitazene (M19) in blood, and *N*-deethyl-5-carboxy-4′-hydroxy etodesnitazene (M2), 5-carboxy-4′-hydroxy etodesnitazene (M6), and *N*-deethyl-5-carboxy etodesnitazene (M16) in urine as metabolite biomarkers of consumption in clinical and forensic settings. Similar to other nitazenes, some of these metabolites might contribute to or prolongate 5-methyl etodesnitazene’s pharmacological effects and toxicity, warranting further research on the drug’s pharmacodynamics.

The fragmentation behavior under mono- and di-protonated conditions in LC-HRMS/MS highlighted the relevance of ionization state for structural elucidation, with di-protonation yielding more informative spectra. This knowledge is critical for developing targeted analytical methods to detect this emerging synthetic opioid and its active metabolites in biological samples. A similar analytical strategy could be applied to other metabolite identification studies on nitazenes, which typically exhibit poor fragmentation patterns in their mono-protonated forms.

## Supplementary Information

Below is the link to the electronic supplementary material.

Supplementary file1 (PDF 892 KB)

Supplementary file2 (PDF 240 KB)

Supplementary file3 (PDF 248 KB)

Supplementary file4 (PDF 254 KB)

## Data Availability

Derived data supporting the findings of this study are available from the corresponding author upon request.

## Abbreviations

AGC: Automatic gain control
ddMS^2^: Data-dependent tandem mass spectrometry
EWS: Early warning system
FullMS: Full-scan mass spectrometry
HESI: Heated electrospray ionization
HRMS: High-resolution mass spectrometry
IT: Injection time
LC-HRMS(/MS): Liquid chromatography-high-resolution (tandem) mass spectrometry
MP: Mobile phase
NCE: Normalized collision energy
NPS: New psychoactive substance
NSO: New synthetic opioid
RT: Retention time
SMILES: Simplified molecular-input line-entry system
SWM: Supplemented Williams’ medium E
UNODC: United Nations Office on Drug and Crime
