# Supplementary material for: 5-Methyl etodesnitazene human metabolism: LC-ESI±-HRMS/MS analysis (mono- and di-protonation) of human hepatocyte incubations and positive biospecimens
Source: Anal Bioanal Chem. 2026 Apr 28;418(12):3679–94. doi: 10.1007/s00216-026-06472-8 (PMC13221404; doi:10.1007/s00216-026-06472-8)

Supplementary Materials. High-resolution tandem mass spectrometry spectra after positive electrospray ionization of 5-methyl etodesnitazene metabolites; [M+H]$^+$, single protonation; [M+2H]$^{2+}$, double protonation. In grey, fragments of some interferents are shown.

M1

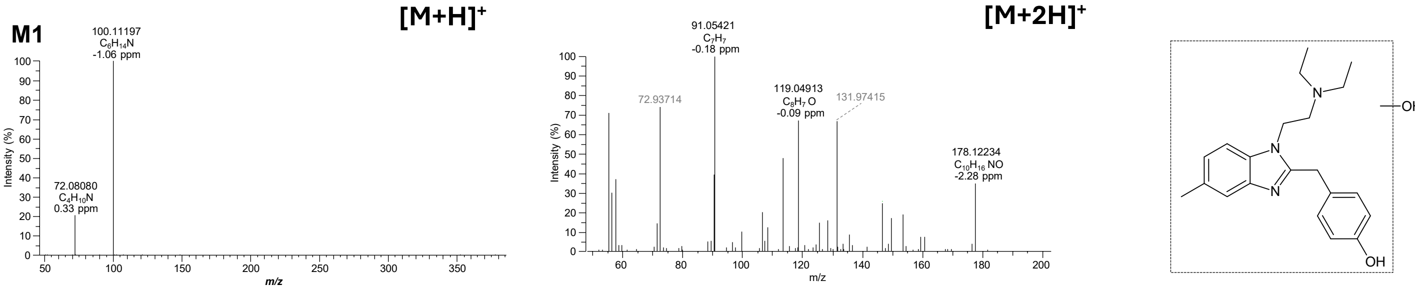

M3

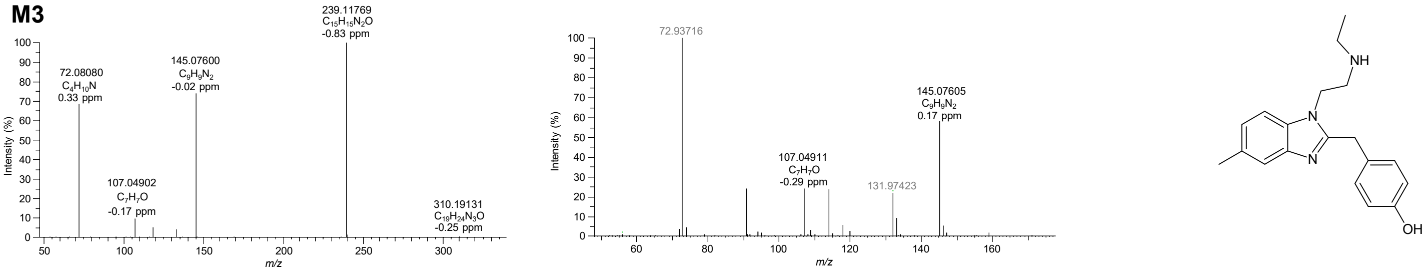

M4

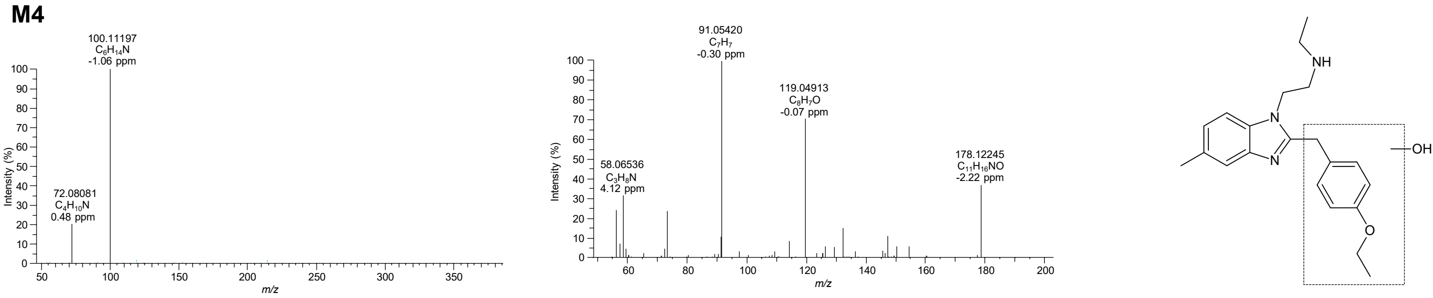

M5

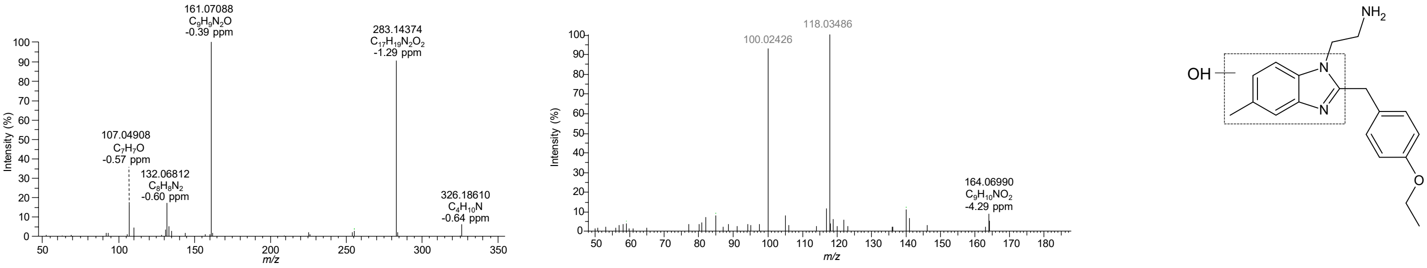

M7

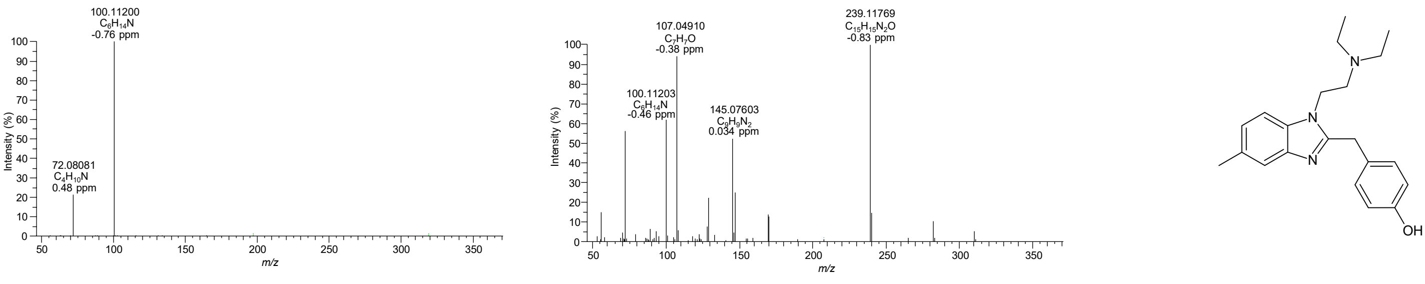

M8

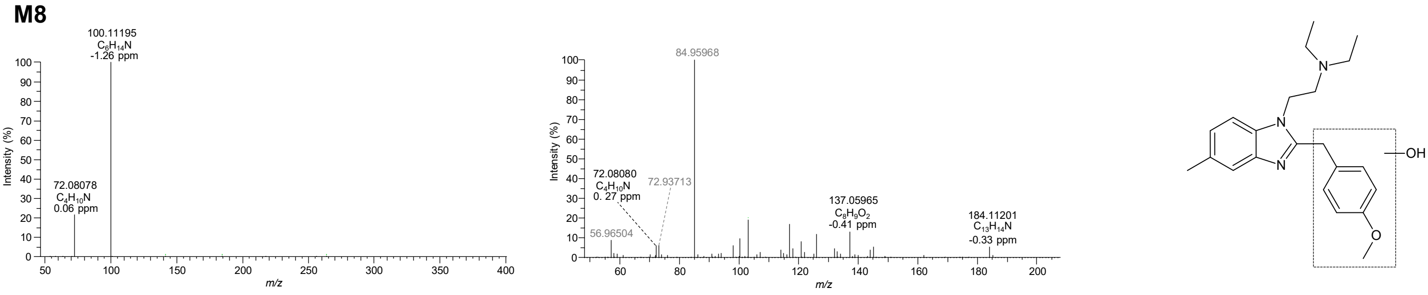

M9

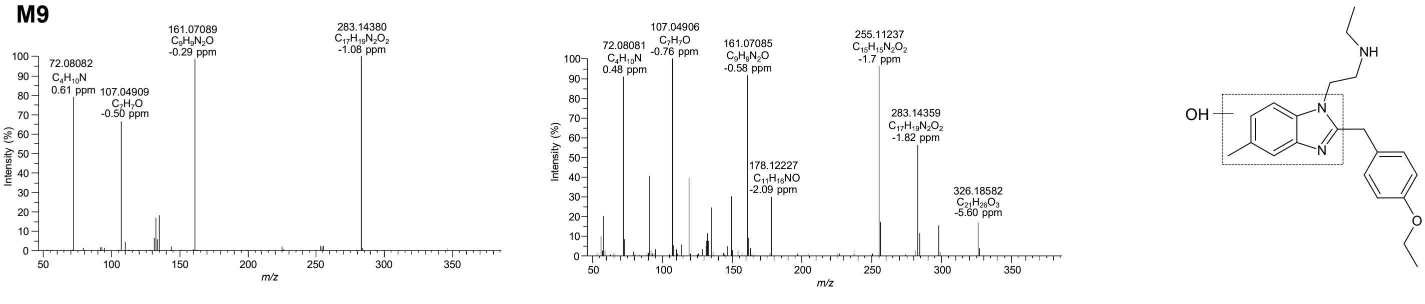

$[M+H]^+$ $[M+2H]^+$

M10

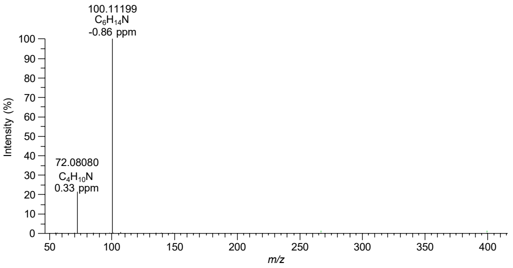

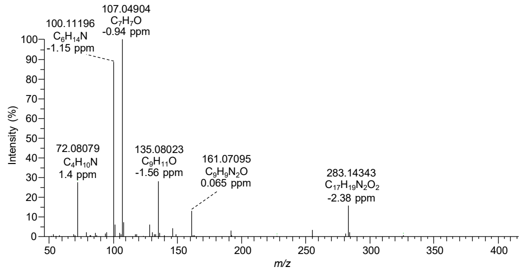

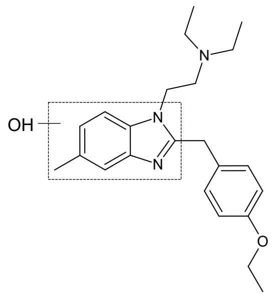

M11

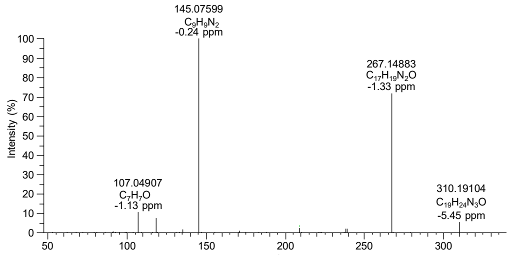

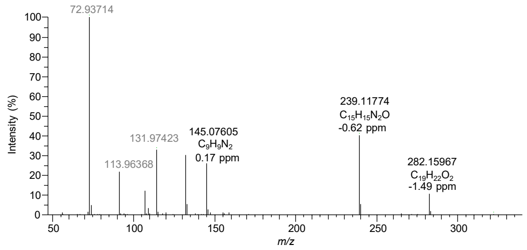

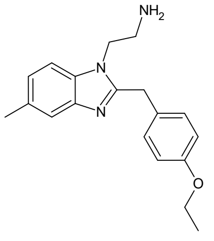

M12

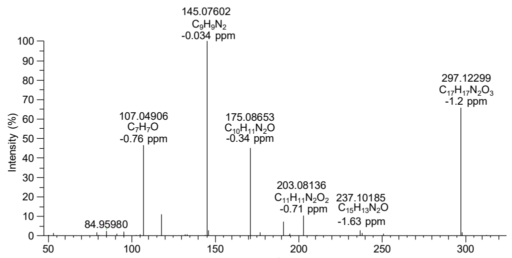

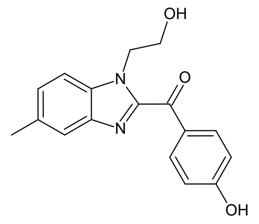

M13U

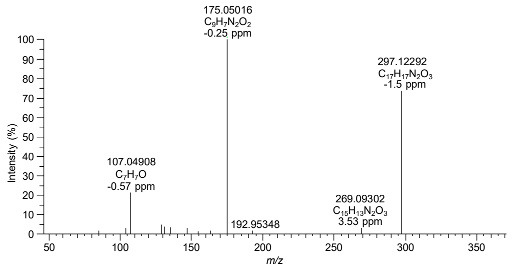

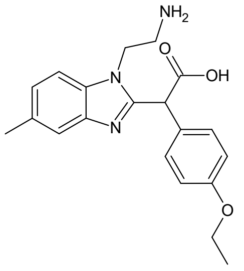

M15

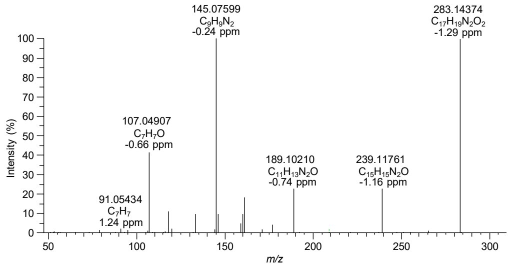

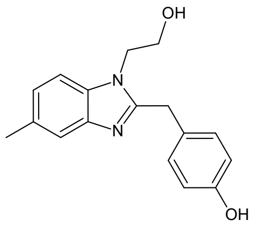

M17U

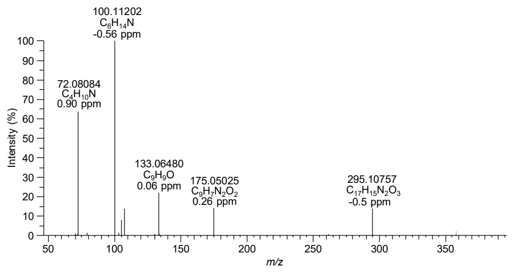

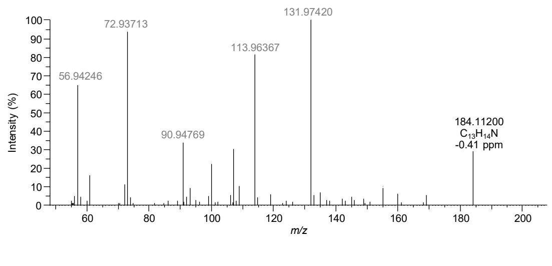

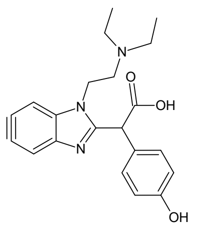

M18

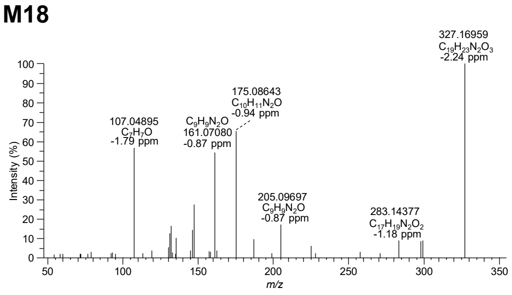

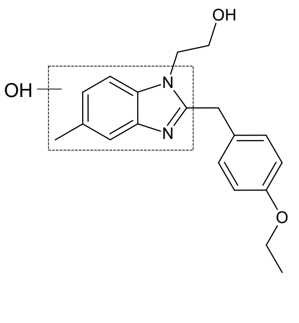

[M+H]$^+$ [M+2H]$^+$

M20

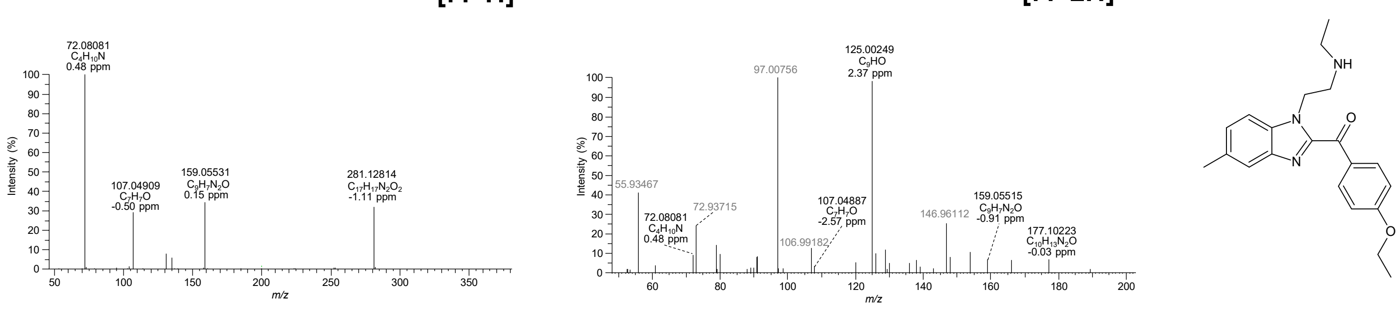

M21

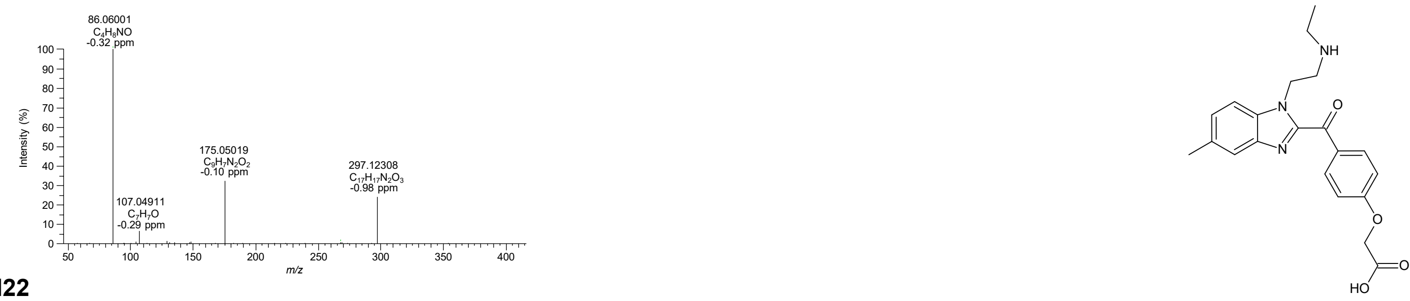

M22

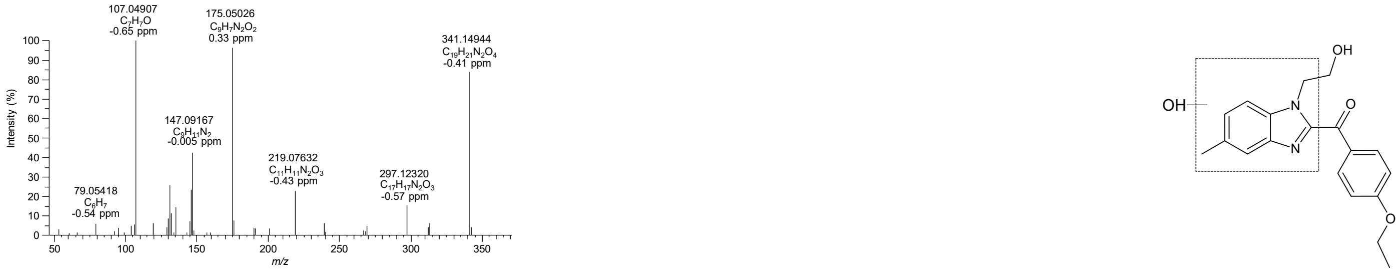

M23

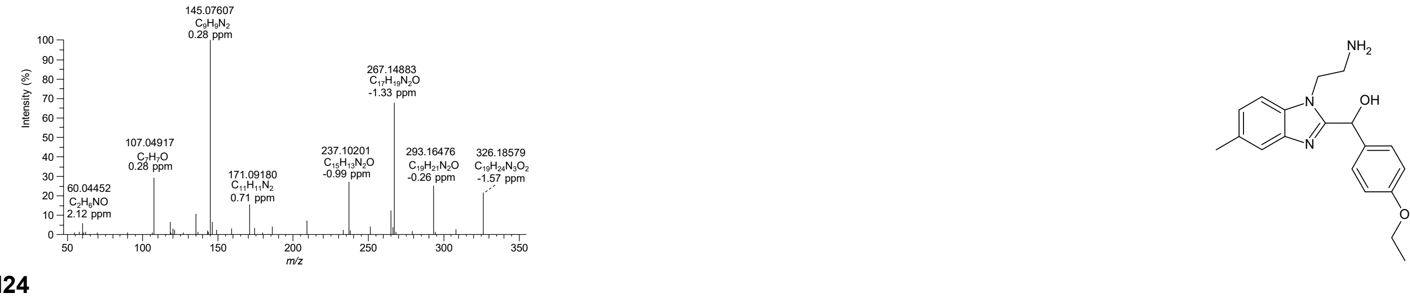

M24

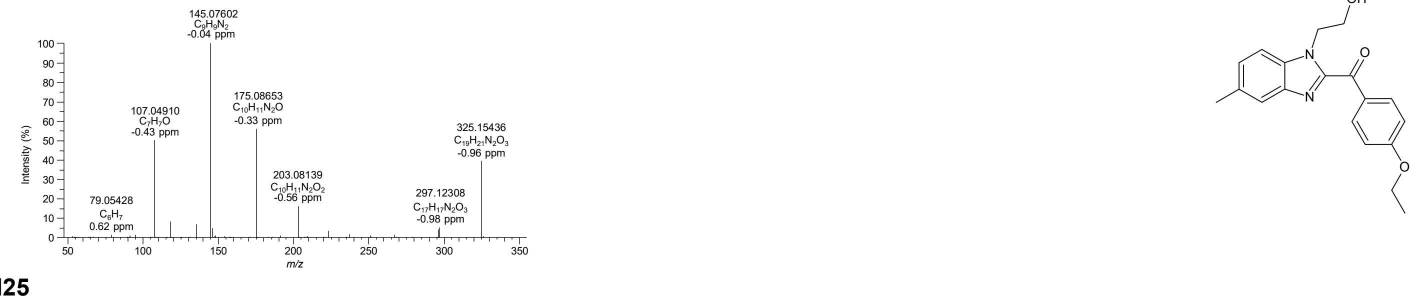

M25

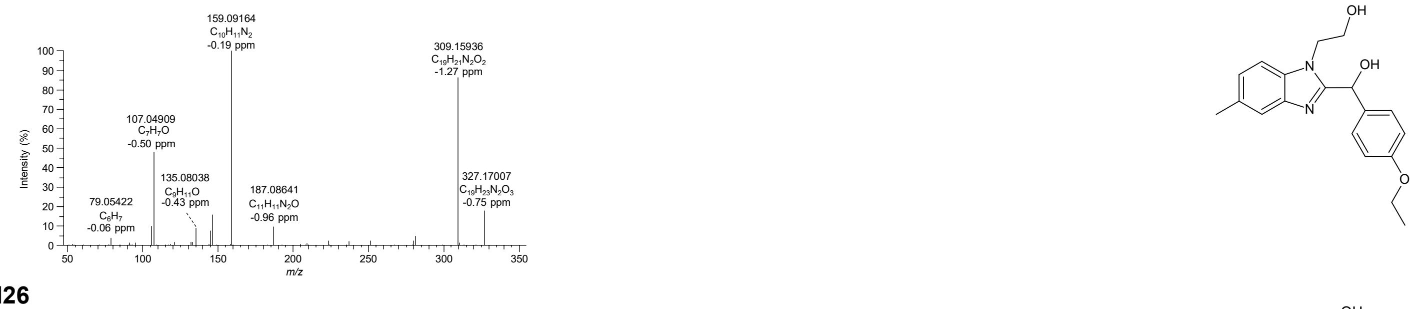

M26

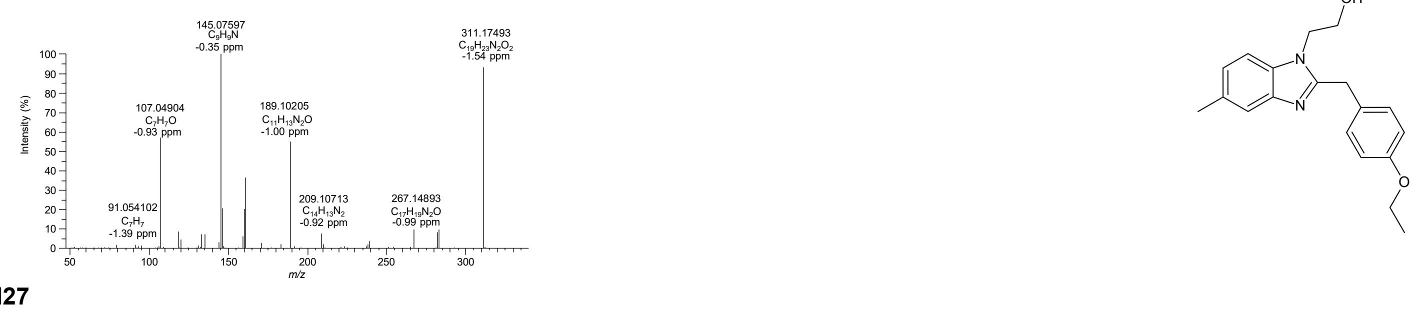

M27

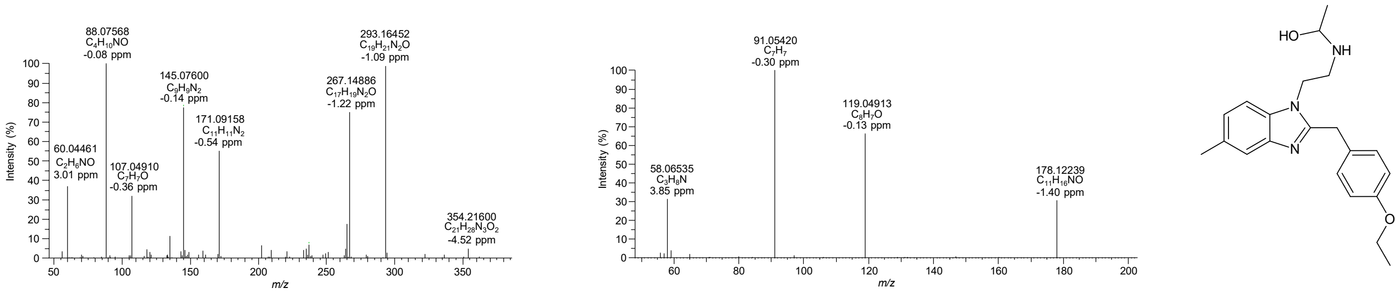

Supplement: Supplementary file 1 — Supplementary file1 (PDF 892 KB) [file 216_2026_6472_MOESM1_ESM.pdf]
