# Supplementary material for: 5-Methyl etodesnitazene human metabolism: LC-ESI±-HRMS/MS analysis (mono- and di-protonation) of human hepatocyte incubations and positive biospecimens
Source: Anal Bioanal Chem. 2026 Apr 28;418(12):3679–94. doi: 10.1007/s00216-026-06472-8 (PMC13221404; doi:10.1007/s00216-026-06472-8)
Supplement: Supplementary file 2 — Supplementary file2 (PDF 240 KB) [file 216_2026_6472_MOESM2_ESM.pdf]

Table S1. Inclusion list used during liquid chromatography-high-resolution tandem mass spectrometry analysis for 5-methyl etodesnitazene metabolite identification.

Transformation	Elemental composition	$[M+H]^+$ $[M+2H]^{2+}$ (*m/z*)	$[M-H]^-$ $[M-2H]^{2-}$ (*m/z*)
Parent	**$\underline{C_{23}H_{31}N_3O}$**	366.2540<br>183.6306	364.2394<br>181.6161
–2C–4H	$C_{21}H_{27}N_3O$	338.2227<br>169.6150	336.2081<br>167.6004
+4C+4H+6O	$C_{27}H_{35}N_3O_7$	514.2548<br>257.6310	512.2402<br>255.6164
+O	$C_{23}H_{31}N_3O_2$	382.2489<br>191.6281	380.2344<br>189.6135
–4C–8H	$C_{19}H_{23}N_3O$	310.1914<br>155.5993	308.1768<br>153.5848
+2C+6O	$C_{25}H_{31}N_3O_7$	486.2235<br>243.6153	484.2089<br>241.6008
–2C–4H+O	$C_{21}H_{27}N_3O_2$	354.2176<br>177.6124	352.2031<br>175.5979
+4C+4H+7O	$C_{27}H_{35}N_3O_8$	530.2497<br>265.6284	528.2351<br>263.6139
+2O	$C_{23}H_{31}N_3O_3$	398.2438<br>199.6255	396.2293<br>197.6109
+6C+8H+7O	$C_{29}H_{39}N_3O_8$	558.2810<br>279.6441	556.2664<br>277.6296
–2H+O	$C_{23}H_{29}N_3O_2$	380.2333<br>190.6202	378.2187<br>188.6057
–2H+2O	$C_{23}H_{29}N_3O_3$	396.2282<br>198.6177	394.2137<br>196.6032
–2C–6H+O	$C_{21}H_{25}N_3O_2$	352.2020<br>176.6046	350.1874<br>174.5901
+4C+2H+7O	$C_{27}H_{33}N_3O_8$	528.2340<br>264.6207	526.2195<br>262.6061
–2C–4H+3O+S	$C_{21}H_{27}N_3O_4S$	418.1795<br>209.5934	416.1650<br>207.5788
–4C–8H+O	$C_{19}H_{23}N_3O_1$	326.1863<br>163.5968	324.1718<br>161.5822
–4C–9H–N+O	$C_{19}H_{22}N_2O_2$	311.1754<br>156.0913	309.1609<br>154.0768
+2C–H–N+7O	$C_{25}H_{30}N_2O_8$	487.2075<br>244.1074	485.1929<br>242.0928
+4O+S	$C_{23}H_{31}N_3O_5S$	462.2057<br>231.6065	460.1912<br>229.5919
–6C–13H–N	$C_{17}H_{18}N_2O$	267.1492<br>134.0782	265.1346<br>132.0637
–4C–9H–N+2O	$C_{19}H_{22}N_2O_3$	327.1703<br>164.0888	325.1558<br>161.0664

–4C–11H–N+2O	$C_{19}H_{20}N_2O_3$	325.1547<br>163.0810	323.1401<br>161.0664
–4C–9H–N+4O+S	$C_{19}H_{22}N_2O_5S$	391.1322<br>196.0697	389.1177<br>174.0552
