# Supplementary material for: 5-Methyl etodesnitazene human metabolism: LC-ESI±-HRMS/MS analysis (mono- and di-protonation) of human hepatocyte incubations and positive biospecimens
Source: Anal Bioanal Chem. 2026 Apr 28;418(12):3679–94. doi: 10.1007/s00216-026-06472-8 (PMC13221404; doi:10.1007/s00216-026-06472-8)
Supplement: Supplementary file 3 — Supplementary file3 (PDF 248 KB) [file 216_2026_6472_MOESM3_ESM.pdf]

Table S2. Compound Discoverer processing settings for generating 5-methyl etodesnitazene putative metabolites**.**

**Phase I reactions**	Dehydration (–2H –O → ø)<br>Desaturation (–2H → ø)<br>*N*-Deethylation (–2C –5H → +H)<br>Hydration (ø → +2H +O)<br>Ketone formation (–O → +2H)<br>Oxidation (ø → +O)<br>Oxidative deamination to alcohol (–2H –N → +H +O)<br>Oxidative deamination to ketone (–3H –N → +O)<br>Reduction (ø → 2H)
**Phase II reactions**	Acetylation (–H → +2C +3H +O)<br>Cysteine conjugation (–H → +3C +6H +N +2O +S)<br>Cysteine-Glycine conjugation (–H → +5C +9H +2N +3O +S)<br>Glucuronide conjugation (–H → +6C +9H +6O)<br>GSH conjugation (–H → +10C +15H +3N +6O +S)<br>Methylation (–H → +C +3H)<br>Sulfation (–H → +H +3O +S)
**Max number of dealkylations**	3
**Max number of phase II reactions**	2
**Max number of all steps**	5
**Adducts**	$[M+H]^+$<br>$[M-H]^-$<br>$[M+2H]^{2+}$<br>$[M-2H]^{2-}$
