# Supplementary material for: 5-Methyl etodesnitazene human metabolism: LC-ESI±-HRMS/MS analysis (mono- and di-protonation) of human hepatocyte incubations and positive biospecimens
Source: Anal Bioanal Chem. 2026 Apr 28;418(12):3679–94. doi: 10.1007/s00216-026-06472-8 (PMC13221404; doi:10.1007/s00216-026-06472-8)
Supplement: Supplementary file 4 — Supplementary file4 (PDF 254 KB) [file 216_2026_6472_MOESM4_ESM.pdf]

Table S3. 5-Methyl etodesnitazene putative metabolites predicted with GLORYx freeware and their prediction score (adjusted score for second-generation metabolites).

ID	Transformation	Elemental composition	Score	Simplified molecular-input line-entry system (SMILES)	Comment
pM1	*N*-Oxidation	$C_{23}H_{31}N_3O_2$	78%	Cc1ccc2n(CC[N+]([O-])(CC)CC)c(nc2c1)Cc1ccc(OCC)cc1	
pM1-1	+ *O*-Dealkylation (aromatic)	$C_{21}H_{27}N_3O_2$	59%	Cc1ccc2n(CC[N+]([O-])(CC)CC)c(nc2c1)Cc1ccc(O)cc1	=PM5-4
pM1-2	+ Aliphatic hydroxylation	$C_{23}H_{31}N_3O_3$	59%	CC(O)Oc1ccc(cc1)Cc1nc2cc(C)ccc2n1CC[N+]([O-])(CC)CC	
pM1-3	+ Carboxylation	$C_{23}H_{29}N_3O_4$	27%	CCOc1ccc(cc1)Cc1nc2cc(ccc2n1CC[N+]([O-])(CC)CC)C(O)=O	=PM7-2
pM1-4	+ Aromatic hydroxylation	$C_{23}H_{31}N_3O_3$	27%	Cc1ccc2n(CC[N+]([O-])(CC)CC)c(nc2c1O)Cc1ccc(OCC)cc1	=PM9-2
pM1-5	+ Benzylic hydroxylation	$C_{23}H_{31}N_3O_3$	27%	CCOc1ccc(cc1)Cc1nc2cc(ccc2n1CC[N+]([O-])(CC)CC)CO	=PM8-3
pM2	Aliphatic hydroxylation	$C_{23}H_{31}N_3O_2$	78%	CCOc1ccc(cc1)Cc1nc2cc(C)ccc2n1CCN(CC)C(C)O	
pM2-1	+ Sulfation (aliphatic hydroxyl)	$C_{23}H_{31}N_3O_5S$	74%	CC(OS(O)(=O)=O)N(CC)CCn1c(nc2cc(C)ccc12)Cc1ccc(OCC)cc1	
pM2-2	+ Aliphatic hydroxylation	$C_{23}H_{31}N_3O_3$	59%	CC(O)Oc1ccc(cc1)Cc1nc2cc(C)ccc2n1CCN(CC)C(C)O	=PM4-2
pM2-3	+ *O*-Dealkylation (aromatic)	$C_{21}H_{27}N_3O_2$	59%	CC(O)N(CC)CCn1c(nc2cc(C)ccc12)Cc1ccc(O)cc1	=PM5-5
pM2-4	+ *O*-Glucuronidation (aliphatic hydroxyl)	$C_{29}H_{39}N_3O_8$	45%	CC(OC1OC(C(O)=O)C(O)C(O)C1O)N(CC)CCn1c(nc2cc(C)ccc12)Cc1ccc(OCC)cc1	
pM2-5	+ Dehydrogenation	$C_{23}H_{29}N_3O_2$	38%	CC(=O)N(CC)CCn1c(nc2cc(C)ccc12)Cc1ccc(OCC)cc1	
pM2-6	+ *N*-Dealkylation	$C_{21}H_{27}N_3O$	38%	Cc1ccc2n(CCNCC)c(nc2c1)Cc1ccc(cc1)OCC	=PM3
pM2-7	+ *N*-Oxidation	$C_{23}H_{31}N_3O_3$	38%	CC(O)[N+]([O-])(CC)CCn1c(nc2cc(C)ccc12)Cc1ccc(OCC)cc1	
pM2-8	+ Aliphatic hydroxylation	$C_{23}H_{31}N_3O_3$	38%	CC(O)(O)N(CC)CCn1c(nc2cc(C)ccc12)Cc1ccc(OCC)cc1	
pM2-9	+ Benzylic hydroxylation	$C_{23}H_{31}N_3O_3$	27%	CC(O)N(CC)CCn1c(nc2cc(ccc12)CO)Cc1ccc(cc1)OCC	=PM8-4
pM2-10	+ Aromatic hydroxylation	$C_{23}H_{31}N_{3O3}$	27%	CC(O)N(CC)CCn1c(nc2c(O)c(C)ccc12)Cc1ccc(OCC)cc1	=PM9-4
pM2-11	+ Carboxylation (benzylic)	$C_{23}H_{29}N_3O_4$	27%	CC(O)N(CC)CCn1c(nc2cc(ccc12)C(O)=O)Cc1ccc(cc1)OCC	=PM7-4
pM3	*N*-Dealkylation	$C_{21}H_{27}N_3O$	78%	Cc1ccc2n(CCNCC)c(nc2c1)Cc1ccc(cc1)OCC	=PM2-6
pM3-1	+ *O*-Dealkylation (aromatic)	$C_{19}H_{23}N_3O$	58%	Oc1ccc(cc1)Cc1nc2cc(C)ccc2n1CCNCC	=PM5-3
pM3-2	+ Aliphatic hydroxylation	$C_{21}H_{27}N_3O_2$	58%	CC(O)Oc1ccc(cc1)Cc1nc2cc(C)ccc2n1CCNCC	=PM4-1
pM3-3	+ Oxidative deamination	$C_{19}H_{22}N_2O_2$	48%	OCCn1c(nc2cc(C)ccc12)Cc1ccc(cc1)OCC	=PM6
pM3-4	+ Aliphatic hydroxylation	$C_{21}H_{27}N_3O_2$	48%	CC(O)NCCn1c(nc2cc(C)ccc12)Cc1ccc(cc1)OCC	
pM3-5	+ Amine hydroxylation	$C_{21}H_{27}N_3O_2$	48%	Cc1ccc2n(CCN(O)CC)c(nc2c1)Cc1ccc(cc1)OCC	
pM3-6	+ *N*-Dealkylation	$C_{19}H_{23}N_3O$	48%	NCCn1c(nc2cc(C)ccc12)Cc1ccc(cc1)OCC	
pM3-7	+ Benzylic hydroxylation	$C_{21}H_{27}N_3O_2$	27%	CCOc1ccc(cc1)Cc1nc2cc(ccc2n1CCNCC)CO	=PM8-5
pM3-8	+ Aromatic hydroxylation	$C_{21}H_{27}N_3O_2$	27%	Cc1ccc2n(CCNCC)c(nc2c1O)Cc1ccc(cc1)OCC	=PM9-3
pM3-9	+ Carboxylation (benzylic)	$C_{21}H_{25}N_3O_3$	27%	OC(=O)c1ccc2n(CCNCC)c(nc2c1)Cc1ccc(cc1)OCC	=PM7-3
pM4	Aliphatic hydroxylation	$C_{23}H_{31}N_3O_2$	76%	CC(O)Oc1ccc(cc1)Cc1nc2cc(C)ccc2n1CCN(CC)CC	
pM4-1	+ *N*-Dealkylation	$C_{21}H_{27}N_3O_2$	58%	CC(O)Oc1ccc(cc1)Cc1nc2cc(C)ccc2n1CCNCC	=PM3-2
pM4-2	+ Aliphatic hydroxylation	$C_{23}H_{31}N_3O_3$	58%	CC(O)Oc1ccc(cc1)Cc1nc2cc(C)ccc2n1CCN(CC)C(C)O	=PM2-2
pM4-3	+ Sulfation(aliphatic hydroxyl)	$C_{23}H_{31}N_3O_5S$	57%	CC(OS(O)(=O)=O)Oc1ccc(cc1)Cc1nc2cc(C)ccc2n1CCN(CC)CC	
pM4-4	+ *O*-Dealkylation (aromatic)	$C_{21}H_{27}N_3O$	48%	Cc1ccc2n(CCN(CC)CC)c(nc2c1)Cc1ccc(O)cc1	=PM5
pM4-5	+ Aliphatic hydroxylation	$C_{23}H_{31}N_3O_3$	48%	CC(O)(O)Oc1ccc(cc1)Cc1nc2cc(C)ccc2n1CCN(CC)CC	
pM4-6	+ Dehydrogenation	$C_{23}H_{29}N_3O_2$	48%	CC(=O)Oc1ccc(cc1)Cc1nc2cc(C)ccc2n1CCN(CC)CC	
pM4-7	+ Aromatic hydroxylation	$C_{23}H_{31}N_3O_3$	26%	CC(O)Oc1ccc(cc1)Cc1nc2c(ccc(C)c2O)n1CCN(CC)CC	
pM4-8	+ Benzylic hydroxylation	$C_{23}H_{31}N_3O_3$	26%	CC(O)Oc1ccc(cc1)Cc1nc2cc(ccc2n1CCN(CC)CC)CO	=PM8-7
pM4-9	+ Carboxylation (benzylic)	$C_{23}H_{29}N_3O_4$	26%	CC(O)Oc1ccc(cc1)Cc1nc2cc(ccc2n1CCN(CC)CC)C(O)=O	=PM7-6
pM5	*O*-Dealkylation (aromatic)	$C_{21}H_{27}N_3O$	76%	Cc1ccc2n(CCN(CC)CC)c(nc2c1)Cc1ccc(O)cc1	=PM4-4
pM5-1	+ *O*-Glucuronidation (aromatic hydroxyl)	$C_{27}H_{35}N_3O_7$	74%	OC(=O)C1OC(Oc2ccc(cc2)Cc2nc3cc(C)ccc3n2CCN(CC)CC)C(O)C(O)C1O	
pM5-2	+ Sulfation (aromatic hydroxyl)	$C_{21}H_{27}N_3O_4S$	72%	Cc1ccc2n(CCN(CC)CC)c(nc2c1)Cc1ccc(cc1)OS(O)(=O)=O	
pM5-3	+ *N*-Dealkylation	$C_{19}H_{23}N_3O$	58%	Oc1ccc(cc1)Cc1nc2cc(C)ccc2n1CCNCC	=PM3-1
pM5-4	+ *N*-Oxidation	$C_{21}H_{27}N_3O_2$	58%	Cc1ccc2n(CC[N+]([O-])(CC)CC)c(nc2c1)Cc1ccc(O)cc1	=PM1-1
pM5-5	+ Aliphatic hydroxylation	$C_{21}H_{27}N_3O_2$	58%	CC(O)N(CC)CCn1c(nc2cc(C)ccc12)Cc1ccc(O)cc1	=PM2-3
pM5-6	+ Aromatic hydroxylation	$C_{21}H_{27}N_3O_2$	35%	Cc1ccc2n(CCN(CC)CC)c(nc2c1)Cc1ccc(O)c(O)c1	
pM6	Oxidative deamination	$C_{19}H_{22}N_2O_2$	38%	OCCn1c(nc2cc(C)ccc12)Cc1ccc(cc1)OCC	=PM3-3
pM6-1	+ Sulfation(aliphatic hydroxyl)	$C_{19}H_{22}N_2O_5S$	35%	OS(=O)(=O)OCCn1c(nc2cc(C)ccc12)Cc1ccc(cc1)OCC	
pM6-2	+ *O*-Glucuronidation (aliphatic hydroxyl)	$C_{25}H_{30}N_2O_8$	32%	OC(=O)C1OC(OCCn2c(nc3cc(C)ccc23)Cc2ccc(cc2)OCC)C(O)C(O)C1O	
pM6-3	+ Aliphatic hydroxylation	$C_{19}H_{22}N_2O_3$	28%	OC(C)Oc1ccc(cc1)Cc1nc2cc(C)ccc2n1CCO	
pM6-4	+ *O*-Dealkylation (aromatic)	$C_{17}H_{18}N_2O_2$	28%	Oc1ccc(cc1)Cc1nc2cc(C)ccc2n1CCO	
pM7	Carboxylation (benzylic)	$C_{23}H_{29}N_3O_3$	35%	CCOc1ccc(cc1)Cc1nc2cc(ccc2n1CCN(CC)CC)C(O)=O	

pM7-1	+ *O*-Glucuronidation (aromatic carboxyl)	$C_{29}H_{37}N_3O_9$	34%	O=C(OC1OC(C(O)=O)C(O)C(O)C1O)c1ccc2n(CCN(CC)CC)c(nc2c1)Cc1ccc(OCC)cc1	
pM7-2	+ *N*-Oxidation	$C_{23}H_{29}N_3O_4$	27%	CCOc1ccc(cc1)Cc1nc2cc(ccc2n1CC[N+]([O-])(CC)CC)C(O)=O	=PM1-3
pM7-3	+ *N*-Dealkylation	$C_{21}H_{25}N_3O_3$	27%	OC(=O)c1ccc2n(CCNCC)c(nc2c1)Cc1ccc(cc1)OCC	=PM3-9
pM7-4	+ Aliphatic hydroxylation	$C_{23}H_{29}N_3O_4$	27%	CC(O)N(CC)CCn1c(nc2cc(ccc12)C(O)=O)Cc1ccc(cc1)OCC	=PM2-11
pM7-5	+ *O*-Dealkylation (aromatic)	$C_{21}H_{25}N_3O_3$	27%	OC(=O)c1ccc2n(CCN(CC)CC)c(nc2c1)Cc1ccc(O)cc1	
pM7-6	+ Aliphatic hydroxylation	$C_{23}H_{29}N_3O_4$	27%	CC(O)Oc1ccc(cc1)Cc1nc2cc(ccc2n1CCN(CC)CC)C(O)=O	=PM4-9
pM8	Benzylic hydroxylation	$C_{23}H_{31}N_3O_2$	35%	CCOc1ccc(cc1)Cc1nc2cc(ccc2n1CCN(CC)CC)CO	
pM8-1	+ Sulfation (aliphatic hydroxyl)	$C_{23}H_{31}N_3O_5S$	33%	CCOc1ccc(cc1)Cc1nc2cc(ccc2n1CCN(CC)CC)COS(O)(=O)=O	
pM8-2	+ *O*-Glucuronidation (aliphatic hydroxyl)	$C_{29}H_{39}N_3O_8$	28%	OC(=O)C1OC(OCc2ccc3n(CCN(CC)CC)c(nc3c2)Cc2ccc(OCC)cc2)C(O)C(O)C1O	
pM8-3	+ *N*-Oxidation	$C_{23}H_{31}N_3O_3$	27%	CCOc1ccc(cc1)Cc1nc2cc(ccc2n1CC[N+]([O-])(CC)CC)CO	=PM1-5
pM8-4	+ Aliphatic hydroxylation	$C_{23}H_{31}N_3O_3$	27%	CC(O)N(CC)CCn1c(nc2cc(ccc12)CO)Cc1ccc(cc1)OCC	=PM2-9
pM8-5	+ *N*-Dealkylation	$C_{21}H_{27N3}O_2$	27%	CCOc1ccc(cc1)Cc1nc2cc(ccc2n1CCNCC)CO	=PM3-7
pM8-6	+ *O*-Dealkylation (aromatic)	$C_{21}H_{27N3}O_2$	27%	Oc1ccc(cc1)Cc1nc2cc(ccc2n1CCN(CC)CC)CO	
pM8-7	+ Aliphatic hydroxylation	$C_{23}H_{31}N_3O_3$	27%	CC(O)Oc1ccc(cc1)Cc1nc2cc(ccc2n1CCN(CC)CC)CO	=PM4-8
pM9	Aromatic hydroxylation	$C_{23}H_{31}N_3O_2$	35%	Cc1ccc2n(CCN(CC)CC)c(nc2c1O)Cc1ccc(OCC)cc1	
pM9-1	+ Sulfation (aromatic hydroxyl)	$C_{23}H_{31}N_3O_5S$	30%	Cc1ccc2n(CCN(CC)CC)c(nc2c1OS(O)(=O)=O)Cc1ccc(OCC)cc1	
pM9-2	+ *N*-Oxidation	$C_{23}H_{31}N_3O_3$	27%	Cc1ccc2n(CC[N+]([O-])(CC)CC)c(nc2c1O)Cc1ccc(OCC)cc1	=PM1-4
pM9-3	+ *N*-Dealkylation	$C_{21}H_{27}N_3O_2$	27%	Cc1ccc2n(CCNCC)c(nc2c1O)Cc1ccc(cc1)OCC	=PM3-8
pM9-4	+ Aliphatic hydroxylation	$C_{23}H_{31}N_3O_3$	27%	CC(O)N(CC)CCn1c(nc2c(O)c(C)ccc12)Cc1ccc(OCC)cc1	=PM2-10
pM9-5	+ *O*-Dealkylation (aromatic)	$C_{21}H_{27}N_3O_2$	27%	Cc1ccc2n(CCN(CC)CC)c(nc2c1O)Cc1ccc(O)cc1	
pM9-6	+ Aliphatic hydroxylation	$C_{23}H_{31}N_3O_3$	27%	CC(O)Oc1ccc(cc1)Cc1nc2c(O)c(C)ccc2n1CCN(CC)CC	
pM9-7	+ *O*-Glucuronidation	$C_{29}H_{39}N_3O_8$	22%	Cc1ccc2n(CCN(CC)CC)c(nc2c1OC1OC(C(O)=O)C(O)C(O)C1O)Cc1ccc(OCC)cc1	
